# Evaluating skeletal muscle dysfunction and recovery in a zymosan model of critical illness in mice

**DOI:** 10.1242/dmm.052712

**Published:** 2026-06-01

**Authors:** Amy J. Bongetti, Annabel Chee, John H. V. Nguyen, Jin D. Chung, Wenlan Li, Kristy Swiderski, Yasmine Ali Abdelhamid, Olav E. Rooyackers, Marissa K. Caldow, Gordon S. Lynch

**Affiliations:** ^1^ Centre for Muscle Research, Department of Anatomy and Physiology, School of Biomedical Sciences, Faculty of Medicine, Dentistry and Health Sciences, The University of Melbourne, Victoria 3010, Australia; ^2^ Intensive Care Unit, The Royal Melbourne Hospital, Victoria 3050, Australia; ^3^ Department of Critical Care, Melbourne Medical School, Faculty of Medicine, Dentistry and Health Sciences, The University of Melbourne, Victoria 3010, Australia; ^4^ Perioperative Medicine and Intensive Care, Karolinska University Hospital, 14186 Stockholm, Sweden; ^5^ Department of Clinical Science, Technology and Intervention, Karolinska Institutet, 14152 Stockholm, Sweden

**Keywords:** Skeletal muscle, Sepsis, Inflammation, Muscle weakness, Muscle wasting, Critical illness, Animal models, Intensive care

## Abstract

Muscle wasting and weakness are common complications associated with critical illness and admission to the intensive care unit (ICU), which contribute to increased mortality and health deficits post discharge. The mechanisms underlying ICU-acquired muscle weakness (ICU-AW) are incompletely understood, and small-animal models can help address this shortfall and provide experimental platforms for devising therapeutic strategies. We used zymosan treatment to induce wasting and weakness in C57BL/6J mice and evaluated recovery of hindlimb muscles and diaphragm on day (D)4, D7, D14 and D28 after induction of critical illness, through extensive physiological and immunohistological analyses. Tibialis anterior muscles from zymosan-treated mice exhibited atrophy and functional impairment at D4 and D7, with recovery at D14. In contrast, the diaphragm exhibited a delay in wasting and recovery from critical illness, with muscle fibre atrophy at D28, despite inflammatory cell infiltration from D4 and transient impairments in respiratory function. The zymosan mouse model provides important insights into mechanisms underlying the recovery from wasting and weakness after critical illness to better understand and treat ICU-AW.

## Teaser

**Summary:** Comprehensive characterisation of a zymosan model of critical illness in mice provides insights into mechanisms underlying muscle wasting and weakness after critical illness and recovery of muscle function.

## INTRODUCTION

Critically ill patients are susceptible to long-term skeletal muscle dysfunction after discharge from the intensive care unit (ICU) (Rousseau et al., 2021; Herridge and Azoulay, 2023), with physical impairments sometimes persisting for up to 5 or 10 years in some patients (Herridge et al., 2011; Meyer-Frießem et al., 2021). The mechanisms underlying the poor recovery from muscle wasting and weakness associated with critical illness are incompletely understood. This lack of information is attributed to the high rates of attrition in clinical studies post hospital discharge (Wilcox and Ely, 2019) and because most research has focussed on the initial insult, such as sepsis, rather than the long-term consequences after critical illness has resolved.

Pre-clinical animal models of critical illness are important for addressing gaps in understanding the mechanistic bases of impaired functional recovery. Most of these models are terminal in nature, such as caecal ligation and puncture (CLP), which involves the isolation, ligation and puncture of the caecum following a laparotomy (Wichterman et al., 1980; Rittirsch et al., 2009; Dejager et al., 2011). Although the CLP model is regarded as the ‘gold-standard’ small-animal model of sepsis (Dejager et al., 2011) and is used extensively in studying critical illness because of its similarities with sepsis in humans, its application can be limited by its invasiveness and difficulty in standardizing illness severity (Bongetti et al., 2025). Furthermore, in rodents, there is a high mortality after CLP, which can limit assessments of long-term functional recovery (Bongetti et al., 2025).

Other models, such as the caecal slurry (CS) model, involving injection of faecal contents into the peritoneal cavity, causes atrophy of the tibialis anterior (TA) muscle in mice 4 days after injection, with recovery of fibre cross-sectional area (CSA) after 14 days (Kingren et al., 2024). Despite the restoration of muscle mass, peak torque of mouse plantar flexors *in vivo* was reduced for up to 70 days after CS injection (Kingren et al., 2024). In this model, long-term deficits in muscle function persisted after resolution of systemic bacterial load (Owen et al., 2019), indicating that skeletal muscle can be compromised irreversibly during recovery from critical illness. Although the CS model facilitates testing of novel therapeutics relevant to critical illness, it does not permit investigation of mechanisms leading to functional recovery, only long-term dysfunction. Although it is less invasive than CLP, it can be difficult to standardize microbial type and concentration and, therefore, disease severity (Bongetti et al., 2025). Hence, there is an urgent need to develop more standardized pre-clinical animal models that can evaluate the mechanisms responsible for muscle wasting and subsequent recovery.

One model for studying recovery from critical illness, muscle atrophy and dysfunction involves induction of peritonitis via intraperitoneal injection of zymosan, a sterile inflammatory agent (Goris et al., 1986; Hill et al., 2015; Rooyackers et al., 1994). Zymosan, a component of the fungal cell wall, activates a systemic inflammatory response through activation of Toll-like receptor 2 (TLR2) (Sato et al., 2003; Volman et al., 2005). Unlike treatments with other endotoxin-like agents, such as lipopolysaccharide (LPS), that do not adequately represent the clinical phenotype of critically ill patients, treatment with a suspension of zymosan in liquid paraffin results in a more representative phenotype due to a more prolonged inflammatory response, compared with LPS administration (Hill et al., 2015; Rooyackers et al., 1994; Volman et al., 2005). Inflammation and critical illness progression in zymosan-treated rats were similar to the inflammatory profiles of septic humanised NSG mice after CLP surgery (Skirecki et al., 2019), with increased plasma IL6 peaking at 6 h and IL10 peaking at 24 h compared with those in vehicle-treated counterparts (Hill et al., 2015). In addition, metabolic characteristics, including oxygen consumption (VO_2_) and carbon dioxide production (VCO_2_), were reduced in zymosan-treated rats after 24 h (Hill et al., 2015), results that were mirrored 30 h after CLP surgery in mice (Oh et al., 2022). Given that zymosan-treated rats also develop multi-organ dysfunction like that in critically ill patients (Hill et al., 2015; Rooyackers et al., 1994; Volman et al., 2005), this model is considered a good candidate for translational studies of recovery from critical illness.

Zymosan-treated rats exhibit a muscle-wasting phenotype followed by subsequent recovery, with atrophy associated with a reduction in peak *in situ* force of the hindlimb plantar flexor muscles on day (D)2 (Hill et al., 2015) and D6 (Minnaard et al., 2005) after zymosan injection, and functional recovery by D14, based on the duration of treadmill running exercise (Hill et al., 2015). However, a more complete characterisation of the skeletal and respiratory muscle phenotype with zymosan administration is required, including the attributes of functional recovery from critical illness. Understanding the relationship between muscle fibre composition and susceptibility to muscle wasting is important for determining the mechanisms underlying muscle impairment in critical illness and potential trajectories for recovery.

To this end, the aim of this study was to establish a zymosan model in mice for evaluating the recovery of muscle wasting and weakness from critical illness. We tested the hypothesis that zymosan administration would impair muscle mass and function at D4 and D7, with a recovery of these parameters by D28. Developing a suitable model of functional recovery would facilitate rigorous evaluation of novel therapeutics for skeletal muscle impairments in critical illness and represents an important contribution to the field.

## RESULTS

C57BL/6J male mice (15-16 weeks) were intraperitonially (i.p.) administered zymosan (0.3 mg/g) suspended in liquid paraffin (25 mg/ml) to assess the impact of critical illness on whole-body metabolism and muscle mass, histopathology and function. To control for the administration of paraffin oil vehicle, mice were also allocated to a vehicle-only group and a control (no-intervention) group. To account for the effect of caloric restriction that occurs in critically ill mice, vehicle-treated mice received food intake in accordance with the eating patterns of the zymosan-treated mice. Peak critical illness occurred (approximately) 2 days after injection of zymosan, with mice showing signs of severe systemic infection, including anorexia, lethargy and piloerection. Recovery from these symptoms was determined to be the resolution of critical illness. Muscles were excised on D4, D7, D14 and D28 after treatment administration. Remnants of paraffin were found in the peritoneal cavity at all timepoints, with adhesions on the liver and diaphragm muscle. Abdominal organs were partially adhered to one another (i.e. the liver was adhered to the diaphragm, or the stomach was adhered to liver) and this appeared to worsen over time. Some of these abnormalities were also evident in vehicle-treated mice but to a lesser extent. None of these adhesions or other abnormalities were identified in mice of the control group.

### Zymosan treatment is a model of critical illness in mice

**Fig. 1.**
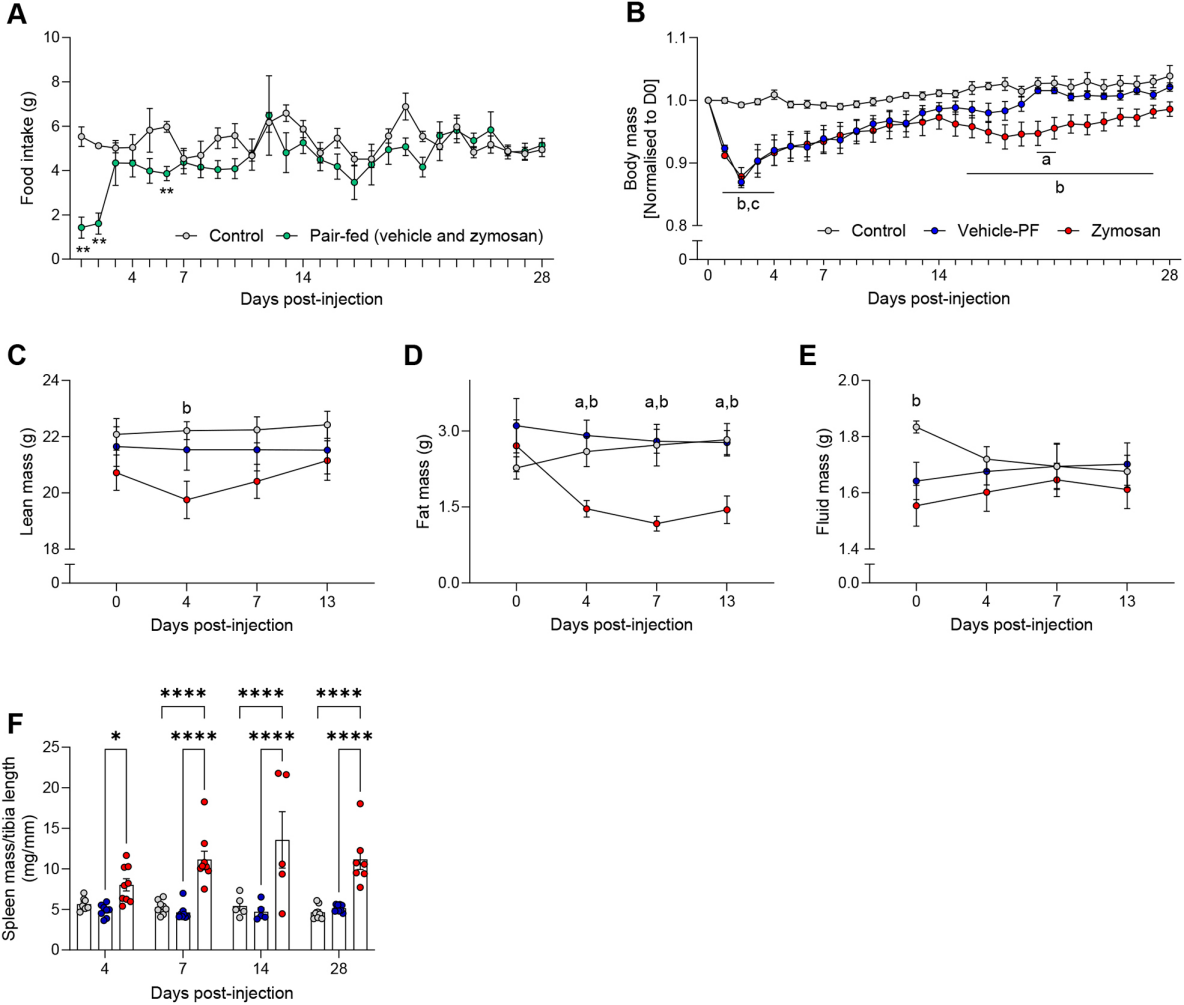
**Zymosan administration causes a reversible critical illness phenotype in mice.** C57BL/6J male mice (15-16 weeks) were allocated to control, vehicle or zymosan groups, with samples collected at day (D)4, D7, D14 and D28 after critical illness induction. (A,B) Zymosan- and vehicle-treated mice exhibited peak reduction in food intake over the first 2 days post treatment (A), associated with a concomitant reduction in body mass compared with that of control mice (B). (C-E) Analysis of lean mass (C), fat mass (D) and fluid mass (E) from whole-body nuclear magnetic resonance time domain imaging. (F) Spleen mass normalized to tibia length was used to assess critical illness phenotype. PF, pair-fed. Data are mean±s.e.m. *n*=5-10 mice/group for A,B,F; *n*=5 mice/group for C-E. Statistical significance was assessed using a mixed-effect analysis (A,B) or a two-way ANOVA with Tukey's multiple comparisons tests (C-F). *P<0.05; **P<0.01; ****P<0.0001. ^a^*P*<0.05, zymosan versus vehicle; ^b^*P*<0.05, zymosan versus control; ^c^*P*<0.05, vehicle versus control.

Zymosan- and pair-fed vehicle-treated mice consumed less food than their control (no-treatment) counterparts (D2; −32%; *P*<0.01, Fig. 1A), which was associated with a concomitant reduction in body mass from D1 to D3 after administration (−7-12%; *P*<0.05, Fig. 1B). However, food intake returned to control levels by D7 and remained unchanged between the three groups to D28 (Fig. 1A). Zymosan-treated mice exhibited a reduction in lean mass at D4 compared to control mice (*P*<0.05, Fig. 1C), with no difference in lean mass at the later timepoints after zymosan administration (D7-D13; Fig. 1C). In comparison, zymosan-treated mice exhibited reduced fat mass relative to both vehicle-treated and control mice from D4, which persisted to D13 (*P*<0.05, Fig. 1D). Despite an increase in fluid mass in control mice relative to both vehicle- and zymosan-treated mice prior to injection (D0), there was no change in fluid mass between the three groups from D4 to D13 (Fig. 1E). In line with an inflammatory response to zymosan administration, spleen mass was increased in zymosan-treated mice compared with that in vehicle-treated mice at D4 (*P*<0.05, Fig. 1F) and with that in both vehicle-treated and control mice from D7 to D28 (*P*<0.0001, Fig. 1F). Liver mass was also higher in zymosan-treated mice compared with that in vehicle-treated mice (*P*<0.0001, Fig. S1A) and control mice at D14 (*P*<0.001, Fig. S1A), and kidney mass was reduced by zymosan administration relative to that in control mice only at D4 (*P*<0.01, Fig. S1B). Heart mass was decreased in both vehicle- and zymosan-treated mice relative to that in control mice at D4 (*P*<0.01, Fig. S1C).

**Fig. 2.**
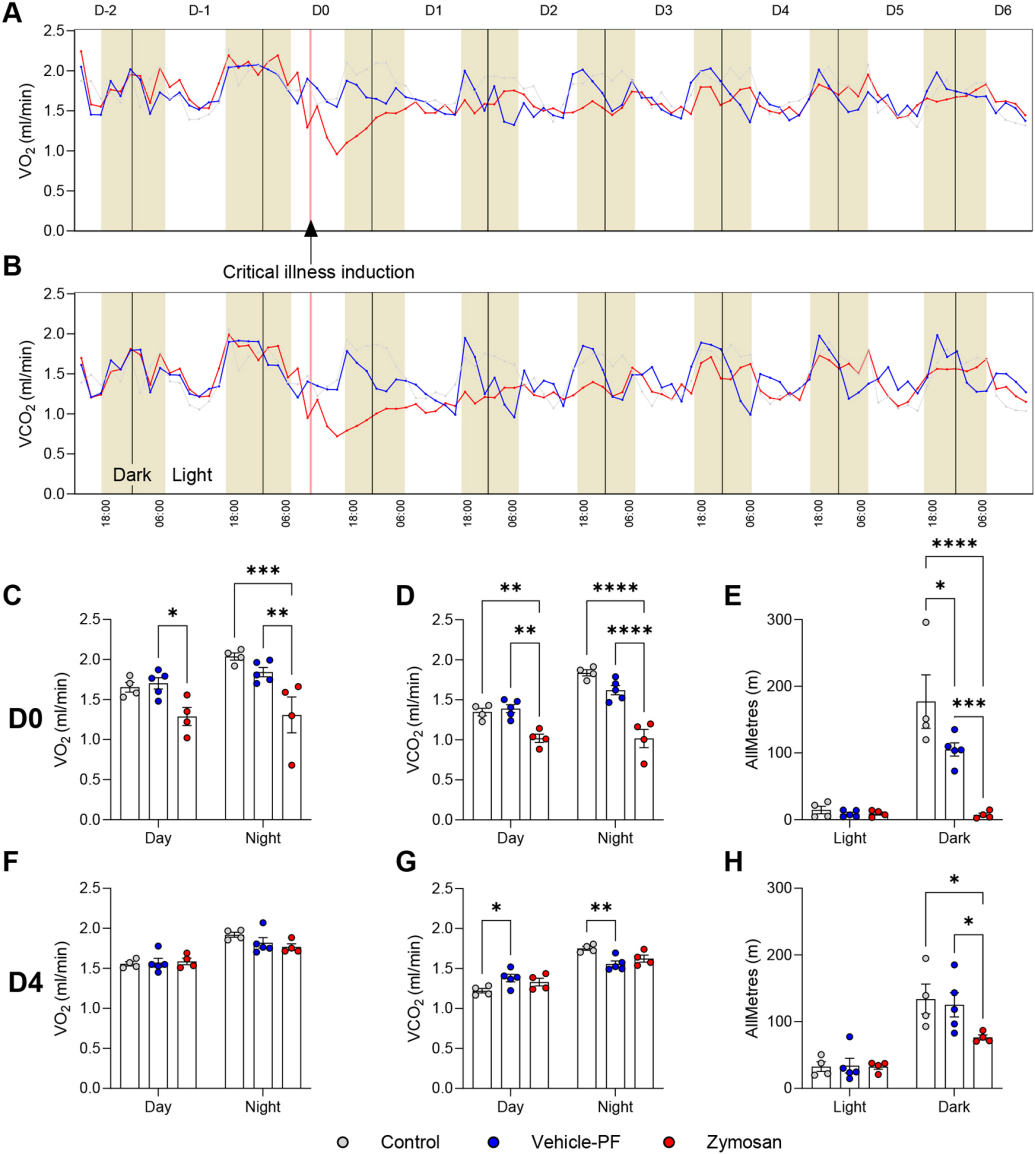
**Metabolic assessments of critically ill mice indicate recovery from zymosan-induced peritonitis.** C57BL/6J male mice (15-16 weeks) were allocated to either control, vehicle or zymosan groups before undergoing whole-body metabolic phenotyping. (A-H) Analysis of oxygen consumption (VO_2_) (A) and carbon dioxide production (VCO_2_) before and after critical illness induction (B); VO_2_ (C), VCO_2_ (D) and ambulation (AllMetres; E) from the Promethion 16-cage system at D0 (C); and VO_2_ (F), VCO_2_ (G) and ambulation (H) at D4. For A,B, data are presented at 2 h intervals. Start, day (D)−2; end, D6; PF, pair-fed. Data are mean±s.e.m. *n*=4-5 mice/group. Statistical significance was determined using a two-way ANOVA with a Tukey's multiple comparisons test. *P<0.05; **P<0.01; ***P<0.001; ****P<0.0001.

To further confirm whether zymosan induced a critical illness phenotype, mice underwent comprehensive metabolic phenotyping (Fig. 2; Figs S2-S6). Compared to vehicle treatment, zymosan treatment reduced VO_2_ (*P*<0.05, Fig. 2A,C), VCO_2_ (*P*<0.01, Fig. 2B,D) and energy expenditure (*P*<0.05, Fig. S2A,B) during the rest (light) phase (06:00-18:00) and the active (dark) phase (18:00-06:00) in the first 24 h after critical illness induction (D0). A significant reduction in ambulation in zymosan-treated mice compared with that in their vehicle-treated counterparts only occurred during the active (dark) phase (*P*<0.001, Fig. 2E) in the first 24 h after critical illness induction. Only minor changes to the respiratory exchange ratio (RER; VCO_2_/VO_2_) were observed, with reductions seen from D0 to D1 in zymosan-treated mice compared to control mice (light and dark phases; *P*<0.05, Fig. S3A-C).

### Recovery from zymosan-induced critical illness in mice

Although the body mass of vehicle-treated pair-fed mice returned to control levels, the body mass of zymosan-treated mice did not return to baseline levels over the 28-day period (*P*<0.05 versus control; Fig. 1B). Recovery of VO_2_ in zymosan-treated mice occurred by D4 compared with that in vehicle-treated mice (Fig. S4A) and by D4 compared with that in control mice (Fig. 2F). Recovery of VCO_2_ in zymosan-treated mice occurred by D3 compared with that in vehicle-treated mice (Fig. S5C) and by D4 compared with that in control mice (Fig. 2G). Recovery of energy expenditure in zymosan-treated mice occurred from D2 compared with that in vehicle-treated mice (Fig. S2D) and by D4 compared with that in control mice (Fig. S2F). Ambulation remained significantly reduced in zymosan-treated mice compared with that in their vehicle-treated counterparts at both D4 (*P*<0.05, Fig. 2H) and D5 (*P*<0.05, Fig. S6D) during the active (dark) phase.

During the rest (light) phase, RER was increased in vehicle-treated mice compared with that in *ad libitum*-fed control mice (D2-D5; *P*<0.01, Fig. S3), highlighting a disruption to diurnal rhythmicity with pair feeding, especially as RER of zymosan-treated mice was equivalent to that of control mice during this period.

### Recovery from muscle wasting and weakness after zymosan-induced critical illness

**Fig. 3.**
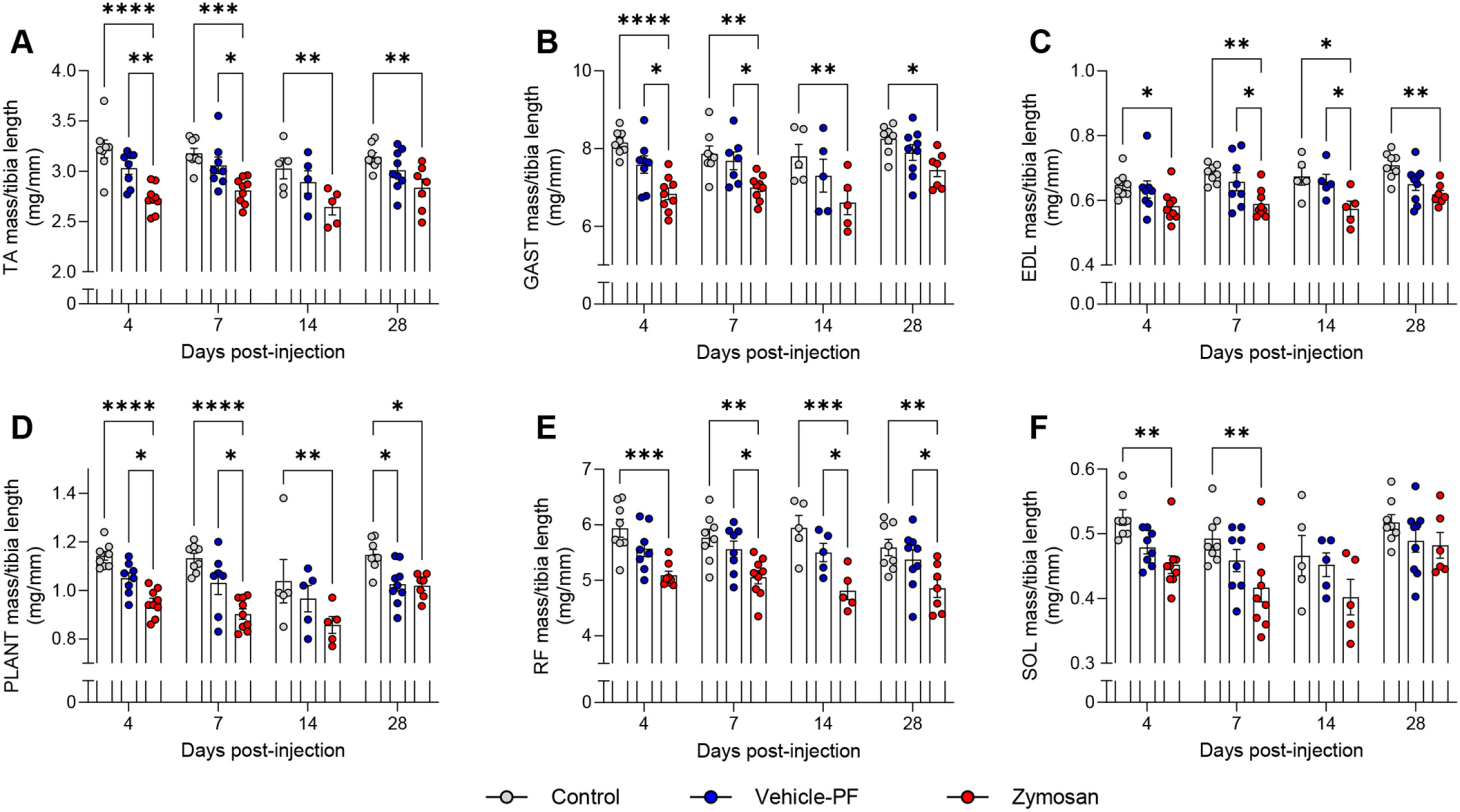
**Hindlimb muscle atrophy and recovery after zymosan-induced critical illness is muscle group dependent.** Muscle mass assessed at D4, D7, D14 and D28 after induction of critical illness for the tibialis anterior (TA; A), gastrocnemius (GAST; B), extensor digitorum longus (EDL; C), plantaris (PLANT; D), rectus femoris (RF; E) and soleus (SOL; F) muscles. PF, pair-fed. Data are mean±s.e.m. *n*=5-10 mice/group, with data for each muscle first normalised to tibia length and then to their respective control groups at each timepoint. Statistical significance was determined using a two-way ANOVA with a Tukey's multiple comparisons test. *P<0.05; **P<0.01; ***P<0.001; ****P<0.0001.

Skeletal muscle health was examined at D4, D7, D14 and D28 after induction of critical illness. TA muscle mass was reduced in zymosan-treated mice at D4 (−10-16%; *P*<0.01, Fig. 3A) and D7 (−8-12%; *P*<0.05, Fig. 3A) compared with that in vehicle-treated and control mice. TA muscle mass was restored to that in vehicle-treated mice by D14, yet it was lower compared with that in control mice (Fig. 3A). A similar pattern of wasting and recovery was observed in the gastrocnemius muscles, with atrophy seen in zymosan-treated mice compared to vehicle-treated mice and control mice at D4 and D7 (−9-16%; *P*<0.05, Fig. 3B). Atrophy of the extensor digitorum longus (EDL) was evident in zymosan-treated mice compared with that in vehicle-treated mice and control mice at D7 and D14 (−10-14%; *P*<0.05, Fig. 3C). Recovery of EDL muscle mass occurred by D28 compared with that in vehicle-treated mice (Fig. 3C). Atrophy of the plantaris muscles was evident at D4 and D7 (−11-20%; *P*<0.05, Fig. 3D) in zymosan-treated mice compared with those in vehicle-treated mice and control mice, which had recovered at D14 compared with those in vehicle-treated mice (Fig. 3D). Wasting of the rectus femoris (RF) muscle was evident at D7 (−9-12%; *P*<0.05, Fig. 3E) in zymosan-treated mice compared with that in vehicle-treated mice and control mice. Despite the recovery from critical illness, atrophy of the RF muscles persisted until D28 in zymosan-treated mice compared with those in vehicle-treated mice and control mice (*P*<0.05, Fig. 3E), suggesting a link between muscle fibre type composition and the recovery from muscle atrophy. Interestingly, soleus muscles did not undergo wasting after zymosan administration compared with those in vehicle-treated mice (Fig. 3F), highlighting that differences in fibre type composition between muscle groups may confer either protection or a greater susceptibility to wasting in this setting.

As zymosan-treated mice experienced a significant reduction in food intake during the critical illness period, vehicle-treated mice received a reduced food intake to match that consumed by the zymosan-treated group to isolate the effect of caloric constriction on muscle mass (Fig. 1A). In a separate study, vehicle-treated mice and control mice were fed *ad libitum* and euthanised 4 days after treatment administration to account for any effect of the vehicle on muscle mass in the absence of caloric restriction (Fig. S7). Compared to pair-fed vehicle-treated mice, *ad libitum*-fed vehicle-treated mice exhibited feeding patterns like those of control mice (Fig. S7A), with no changes to body mass observed (Fig. S7B). Organ and muscle masses were not affected by vehicle treatment alone (Table S1). Vehicle treatment did not affect fibre diameter or fibre number in the TA, RF, soleus or diaphragm muscles (Fig. S7C-F).

### Preferential wasting of fast, glycolytic fibres influences the extent of muscle atrophy in zymosan-induced critical illness

**Fig. 4.**
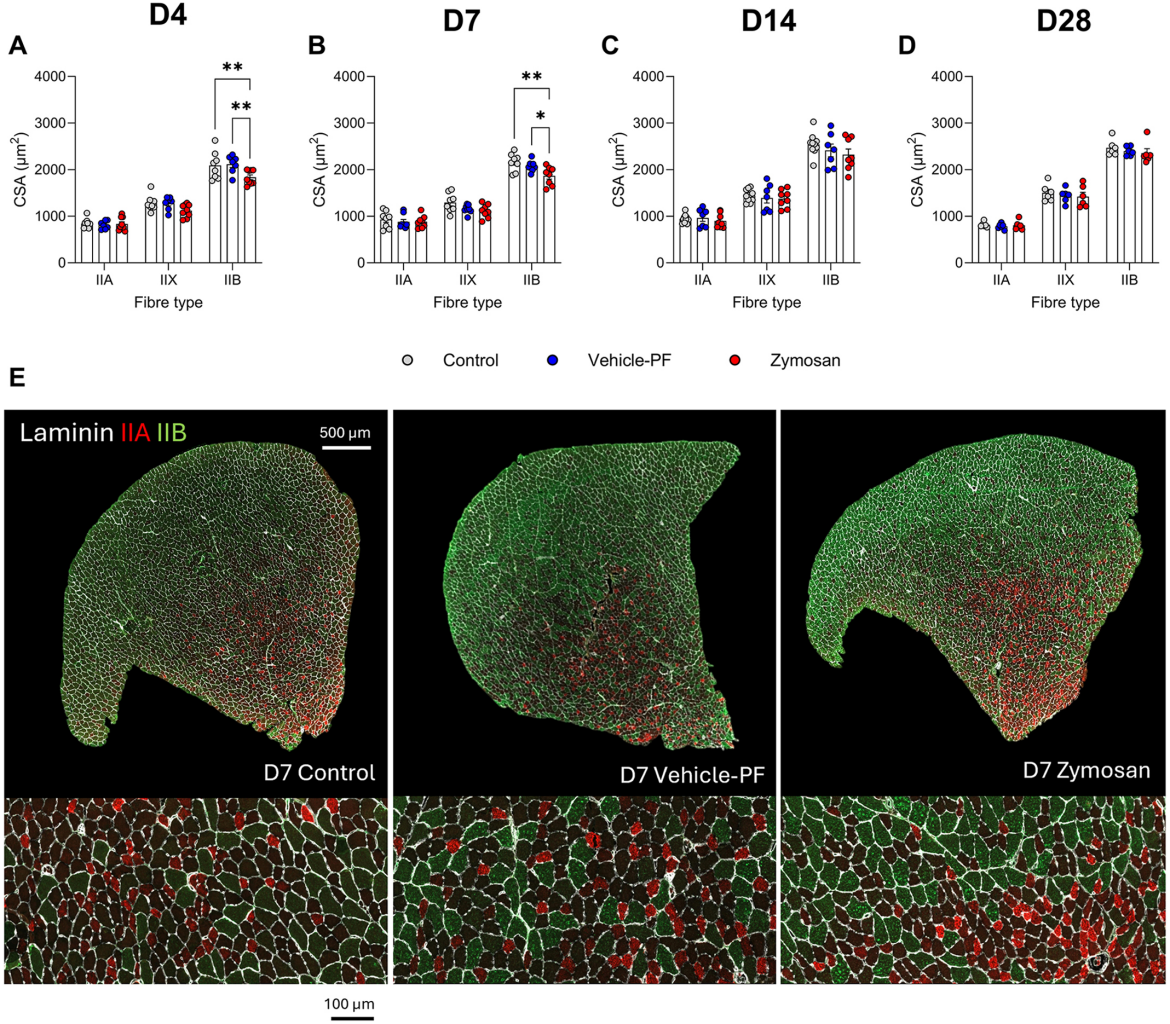
**Analysis of fibre size and fibre types within the TA muscles of mice after zymosan-induced critical illness.** C57BL/6J male mice (15-16 weeks) were allocated to control, vehicle or zymosan groups, with samples collected at D4, D7, D14 and D28 after critical illness induction. (A-D) Cross-sectional area (CSA) by fibre type was assessed at D4 (A), D7 (B), D14 (C) and D28 (D), respectively. (E) Representative images of the TA muscle at D7 showing laminin (white) IIA muscle fibres (red) and IIB muscle fibres (green). PF, pair-fed. Data are mean±s.e.m. *n*=6-10 mice/group. Statistical significance was determined using a two-way ANOVA with a Tukey's multiple comparisons test. *P<0.05; **P<0.01.

**Fig. 5.**
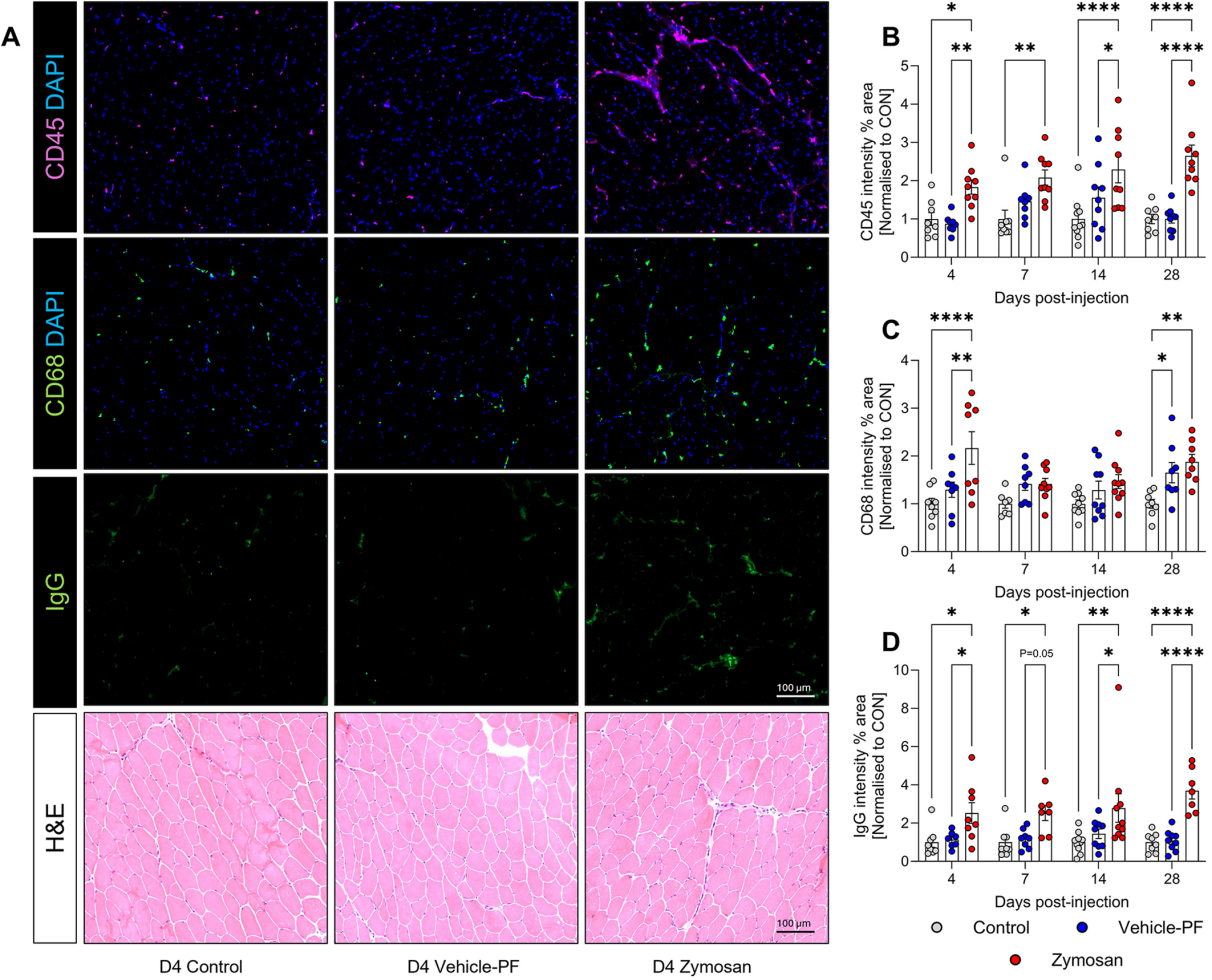
**Inflammatory infiltration in TA muscles of mice after zymosan-induced critical illness.** (A-D) Assessment of CD45^+^ (A,B) and CD68^+^ (A,C) inflammatory cells, and IgG infiltration (A,D) was determined via immunofluorescence of stained TA muscle samples at D4, D7, D14 and D28 after zymosan administration. Representative images of Haematoxylin and Eosin (H&E) staining of TA muscles revealing no structural damage at D4 (A). All representative images shown are from D4. PF, pair-fed; CON, control. Data are mean±s.e.m. *n*=7-10 mice/group. Statistical significance was determined using a two-way ANOVA with a Tukey's multiple comparisons test. *P<0.05; **P<0.01; ****P<0.0001.

Preferential wasting of fast, glycolytic type IIB fibres in the TA muscles was evident at D4 (−12-13%; *P*<0.01, Fig. 4A) and D7 (−10-14%; *P*<0.05, Fig. 4B,E) after zymosan administration compared with that in vehicle-treated and control mice. This was associated with overall muscle wasting at these time points (*P*<0.05, Fig. 3A). Restoration of type IIB fibre CSA was evident at D14 (Fig. 4C) and D28 (Fig. 4D), coinciding with the restoration of muscle mass (Fig. 3A). No changes to muscle fibre number or muscle fibre type proportions (D4-D28; Fig. S8) were observed. Although fibre CSA was restored by D14, infiltration of CD45^+^ immune cells was significantly higher in the TA muscles of zymosan-treated mice compared with that in vehicle-treated and control mice (D4, D14, D28; *P*<0.05, Fig. 5A,B). In comparison, infiltration of monocytes (CD68^+^ cells), a subset of CD45^+^ immune cells, was increased in TA muscles only at D4 after zymosan administration compared with that in vehicle-treated mice (*P*<0.05, Fig. 5A,C). In addition, IgG accumulation was increased in TA muscles at D4, D14 and D28 after zymosan administration (*P*<0.05, Fig. 5A,D) and a trend for increased IgG was seen at D7 (*P*\=0.05). Taken together, these findings suggest a prolonged inflammatory response in skeletal muscle after zymosan administration. Despite inflammatory cell infiltration and muscle atrophy, there was no obvious changes to muscle fibre architecture [as seen by Haematoxylin and Eosin (H&E) staining; Fig. 5A].

Despite prolonged wasting of the RF muscles from D4 to D28 (Fig. 3E), type IIB fibre atrophy was evident only at D4 (*P*<0.01, Fig. S9A) and D7 (−15-24%; *P*<0.01, Fig. S9B) in zymosan-treated mice compared with that in vehicle-treated mice and control mice. Restoration of type IIB fibre CSA occurred at D14 (Fig. S9C) in zymosan-treated mice compared with that in vehicle-treated mice, preceding the clearance of monocyte infiltration from the muscle (CD68^+^ cells; D4, D7, *P*<0.05, Fig. S10). No changes in fibre type proportion (D4-D28; Fig. S9) or fibre number were observed in the RF muscles (D4-D28; Fig. S9). Despite the overall atrophy of RF muscles at D14 and D28, fibre atrophy was not evident, but there was an increase in monocyte infiltration at D28 in zymosan-treated mice compared with that in vehicle-treated and control group mice (CD68^+^ cells, *P*<0.05, Fig. S10).

The soleus muscle, comprising a higher proportion of slow, oxidative fibres, was protected from wasting (Fig. S11). Despite no changes in muscle fibre size, fibre proportions or fibre number (Fig. S11), monocyte infiltration was increased at D7 in zymosan-treated mice compared with that in vehicle-treated and control mice, with a clearance of immune cells from the muscle by D14 (CD68^+^ cells; *P*<0.001, Fig. S12).

As with the TA, atrophy of the RF (Fig. S10B) and soleus muscles was not associated with muscle fibre damage (Fig. S12B).

### Functional recovery of TA muscles after induction of critical illness

**Fig. 6.**
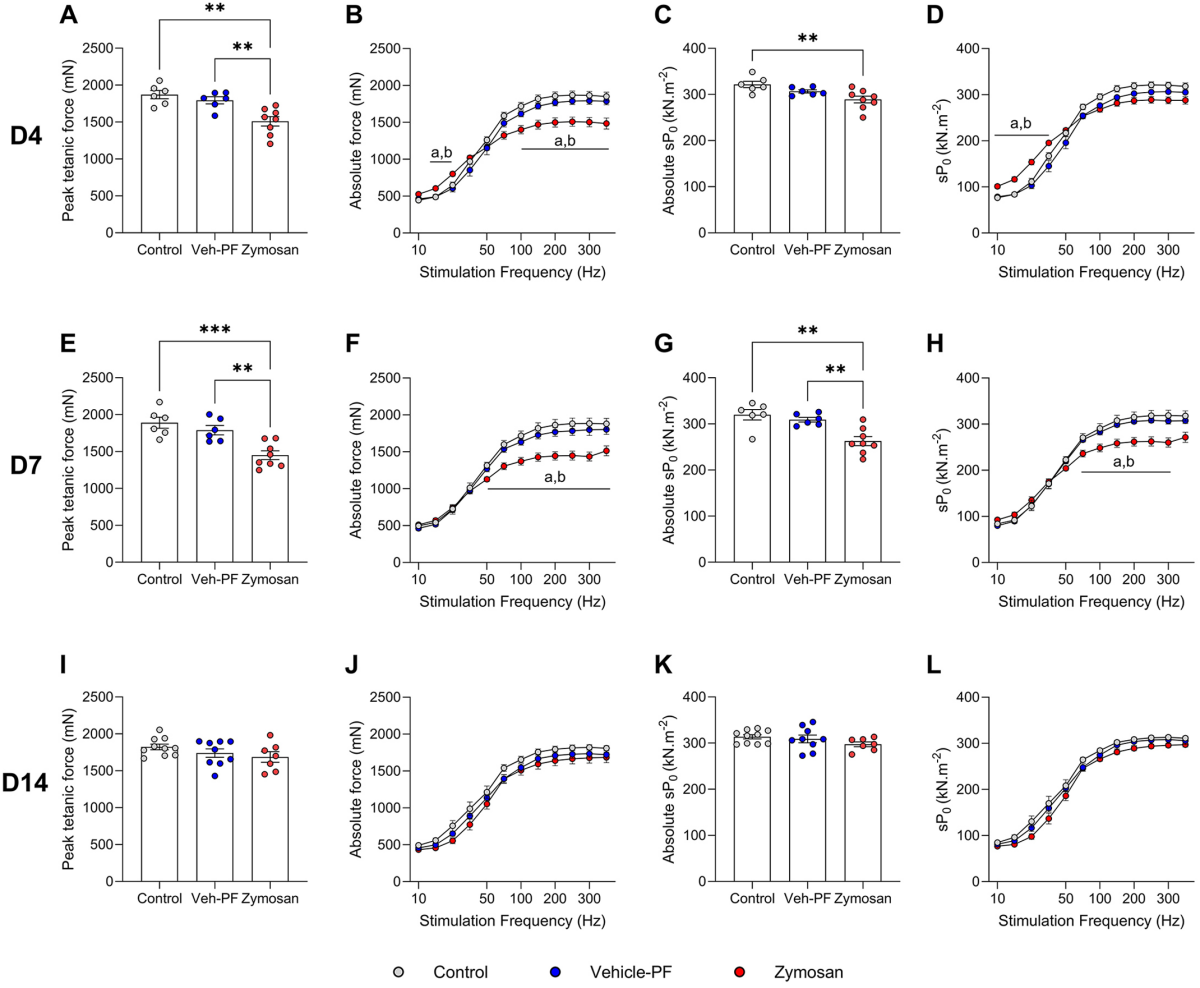
**Recovery of TA muscle function at 14 days after critical illness induction.** (A-L) Muscle function [peak tetanic force, absolute force, peak specific force and specific force (sP_0_)] was assessed at D4 (A-D), D7 (E-H) and D14 (I-L). Force frequencies were assessed at increasing stimulation frequencies (10-350 Hz). Peak specific force (tetanic force normalised to overall muscle CSA). PF, pair-fed; Veh, vehicle. Data are mean±s.e.m. *n*=6-10 mice/group. Statistical significance was determined using a one-way ANOVA (A,C E,G,I,K), a mixed-effect analysis (B,D,F,H) or a two-way ANOVA with Tukey's multiple comparisons test (J,L). **P<0.01; ***P<0.001. ^a^*P*<0.05, zymosan versus vehicle; ^b^*P*<0.05, zymosan versus control.

Isometric contractile properties of TA muscles were assessed *in situ* at D4, D7 and D14 after zymosan administration. Peak tetanic force was reduced in muscles from zymosan-treated mice at D4 (*P*<0.05, Fig. 6A,B) and D7 (*P*<0.05, Fig. 6E,F) compared with that in muscles from vehicle-treated and control mice. Normalised peak (specific) force was reduced in zymosan-treated mice at D4 compared with that in control mice (*P*<0.01, Fig. 6C,D), and at D7 compared with that in vehicle-treated and control mice (*P*<0.05, Fig. 6G,H), indicating that the functional deficits were attributed to factors other than just muscle atrophy. Interestingly, at D4, absolute force at submaximal stimulation frequencies (20-30 Hz, *P*<0.05, Fig. 6B) and specific force were higher in zymosan-treated mice compared with those in vehicle-treated mice and control mice (10-40 Hz, *P*<0.05, Fig. 6D), with the muscle contractile characteristics resembling those of a slow phenotype. Functional recovery was evident by D14 (Fig. 6I-L). Other parameters relating to muscle function were unchanged (Fig. S13), except for an increase in half relaxation time in zymosan-treated mice compared with that in vehicle-treated mice (D4, *P*<0.05, Fig. S13B).

As fatigue is a key factor in the health of critically ill patients, we assessed muscle fatigue from repeated contractions *in situ*. TA muscles from zymosan-treated mice were weaker than those from control mice at the start of the fatigue protocol, and throughout the repeated assessments of force and into recovery (D4-D7; *P*<0.05, Fig. S14A,C). However, the fatigue response was not different between treatments based on specific force (Fig. S14B,D,F).

### Differential wasting of diaphragm and hindlimb muscles after zymosan-induced critical illness

**Fig. 7.**
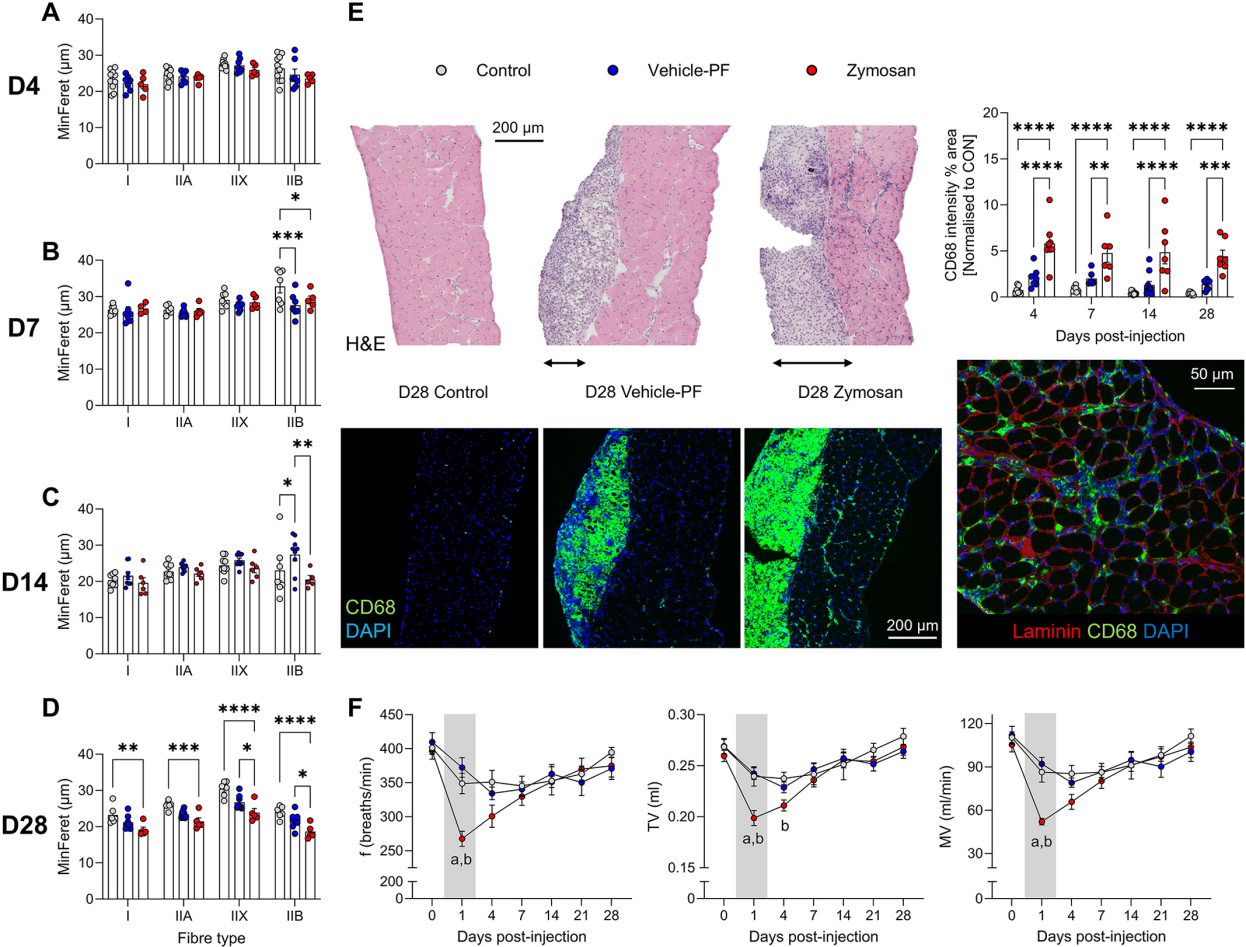
**Differential wasting in the diaphragm after zymosan-induced critical illness does not cause functional deficits under resting conditions.** (A-D) The diaphragm was excised intact at D4 (A), D7 (B), D14 (C) and D28 (D) after critical illness induction, with a portion taken for histological analysis. For each timepoint examined, data are presented for muscle fibre diameter (MinFeret) and fibre proportions. (E) Representative images of H&E staining and inflammatory infiltration via CD68 immunofluorescence at D28. Double arrowheads indicate microgranulomas on the abdominal side of the diaphragm. The enlarged image shows immune (CD68^+^ cells; green) and nuclei (blue) infiltrate between the myofibres of the diaphragm (laminin; red). Quantification of CD68 intensity shown in the graph. (F) Respiratory dysfunction was examined at D−1 (baseline), D1, D4, D7, D14, D21 and D28 after critical illness induction. Respiratory frequency (*f*; left panel), tidal volume (TV; middle panel) and mechanical ventilation (MV; right panel) were affected only at the onset of critical illness (D1). CON, control; MinFeret, myofibre diameter; PF, pair- fed. Data are mean±s.e.m. *n*=6-10 mice/group. Statistical significance was determined using a two-way ANOVA with a Tukey's multiple comparisons test. *P<0.05; **P<0.01; ***P<0.001; ****P<0.0001. ^a^*P*<0.05, zymosan versus vehicle; ^b^*P*<0.05, zymosan versus control.

As respiratory dysfunction is common in critically ill patients, we examined muscle wasting in the diaphragm. Unlike in hindlimb muscles, the diaphragm exhibited a delay in wasting after zymosan administration and a subsequent delayed recovery from critical illness (Fig. 7A-D). Muscle fibre atrophy in the diaphragm of zymosan-treated mice was evident at D28, with significant atrophy of type IIB (*P*<0.05, Fig. 7D) and type IIX (*P*<0.05, Fig. 7D) fibres compared with that in vehicle-treated and control mice. This wasting occurred without alterations in fibre proportions, indicating an overall fibre atrophy rather than a loss of muscle fibres (Fig. S15).

The differences between hindlimb muscles and the diaphragm may be attributed to the presence of progressively worsening microgranulomas adhering to the abdominal side of the diaphragm muscle of zymosan-treated mice over the 28 days (Fig. S16). This was associated with evidence of muscle fibre damage and infiltration at D28 in zymosan-treated mice compared to control mice (Fig. 7E). Monocyte infiltration between muscle fibres within the diaphragm was evident from D4 until D28 (CD68^+^ cells; *P*<0.05, Fig. 7E). Microgranulomas were also adhered to the diaphragm of vehicle-treated mice at D28 (Fig. 7E; Fig. S16D), but this was not associated with the infiltration of inflammatory cells into the diaphragm (CD68^+^ cells; Fig. 7E). It is likely that the diaphragm was more severely affected because of its proximity to the induction of critical illness from the zymosan injection directly into the peritoneal cavity.

### Structural deficits in the diaphragm do not cause overall respiratory dysfunction

Based on histological changes within the diaphragm that might be expected to affect functional capacity and respiratory performance, we assessed basal respiratory function through whole-body plethysmography (WBP). Overall respiratory function was evaluated prior to the induction of critical illness (day −1; baseline) and at D1, D4, D7, D14, D21 and D28 after zymosan administration.

At the peak phase of critical illness (D1), zymosan-treated mice exhibited respiratory depression, with reductions in respiratory frequency (*f*; *P*<0.01, Fig. 7F, left panel), tidal volume (TV; *P*<0.05, Fig. 7F, middle panel) and subsequent minute ventilation (MV; *P*<0.01, Fig. 7F, right panel) compared with those in vehicle-treated and control mice. At D4, TV was reduced in zymosan-treated mice compared with that in control mice (*P*<0.05, Fig. 7F, middle panel).

Peak inspiratory flow (PIF) was decreased in zymosan-treated mice (D1, *P*<0.001, Fig. S17A, left panel), with a concomitant increase in inspiratory time (T_i_; D1, *P*<0.001, Fig. S17A, middle panel) and a decrease in end inspiratory pause (EIP; the time taken to transition from inspiration to expiration) compared with those in vehicle-treated and control mice (D1, *P*<0.05, Fig. S17A, right panel). Reduced peak expiratory flow (PEF) was observed in zymosan-treated mice compared with that in vehicle-treated and control mice (D1, *P*<0.01, Fig. S17B, left panel). PEF was reduced at D4 in zymosan-treated mice compared with that in control mice (*P*<0.05, Fig. S17B, left panel), with reductions in the rate of perfusion (R_pef_) at D1 in zymosan-treated mice compared with that in vehicle treated mice (*P*<0.05, Fig. S17C, left panel). R_pef_ was increased in vehicle-treated mice compared with that in control mice at D1 (*P*<0.05, Fig. S17C, left panel).

No changes in the pause between early and late expiration (Fig. S17D, left panel) or enhanced pause (P_enh_; Fig. S17D, middle panel), indicative of bronchoconstriction, were evident in zymosan-treated mice following the induction of critical illness, indicating that this was not an obstructive condition. All parameters in zymosan-treated mice returned to baseline levels by D7, indicating a recovery from critical illness. The only exception was for relaxation time (TR), which was elevated in vehicle-treated mice compared with that in control mice at D28 (*P*=0.05, Fig. S17C, right panel). There was no association between the histological appearance of the diaphragm and basal respiratory performance in the zymosan-treated mice compared with vehicle-treated mice and control mice when assessed under resting conditions.

## DISCUSSION

Understanding the mechanisms underlying the poor recovery from ICU-AW represents an important unmet clinical need, given the current lack of treatments for patients experiencing long-term physical impairment post discharge. Most studies focus on the initial insult, such as sepsis, rather than the long-term consequences after critical illness has resolved. Animal models of critical illness can help address these gaps in understanding muscle dysfunction, although most are generally terminal in nature rather than platforms for evaluating functional recovery. We utilised a zymosan model in mice to assess the recovery from critical illness and showed a restoration of muscle mass and functional capacity depending on muscle fibre composition. Recovery from critical illness occurred around D3, with muscle atrophy persisting from D4 until D7. The model facilitated functional assessments for longer than previous studies (Hill et al., 2015; Rooyackers et al., 1994; Minnaard et al., 2005) and represents the first study to investigate recovery from critical illness 28 days after zymosan administration. The findings support zymosan treatment of mice being a model for both the acute critical phase and prolonged recovery, with recovery influenced by muscle fibre composition and the proximity to the site of zymosan injection. The model is a suitable platform for the rigorous evaluation of novel interventions for muscle impairments in critical illness and ICU-AW. This is especially important given the difficulty in studying patients during recovery, because of their heterogeneity and as many are too sick to participate in long-term recovery studies.

The trajectory of recovery in the zymosan model of critical illness in mice was similar to that for rats (Hill et al., 2015; Rooyackers et al., 1994) and suitable for investigating therapeutic interventions. Recovery from critical illness was based on the resolution of illness symptoms, ambulation and food intake. Food intake returned to normal by D3 unlike in other studies (in rats), in which food intake only returned to ∼80% of normal levels over a 12-day period (Rooyackers et al., 1994; Minnaard et al., 2005), a discrepancy likely attributed to the higher dose used and differences in metabolism between mice and rats.

Depressions in VO_2_, VCO_2_ and energy expenditure in zymosan-treated mice returned to baseline by D4. Milder doses of zymosan in rats resulted in similar reductions in VO_2_ and VCO_2_ compared with those in vehicle-treated and control mice, with recovery at D5-D6 (Hill et al., 2015). Hypercatabolism with increased oxygen consumption and resting metabolic rate has been reported in patients with sepsis, although it is less common with better sedation and pain management (Kreymann et al., 1993). Unlike studies in CS mice, in which the hypometabolic phenotype was associated with reduced food intake alone (Pierre et al., 2023), inclusion of the pair-fed vehicle-treated group for metabolic investigations showed that reduced food intake alone was not responsible for the metabolic disturbances in zymosan-treated mice.

Similar to previous studies showing diurnal disruptions in metabolism with zymosan administration (Hill et al., 2015), we showed metabolic disruptions prior to recovery from critical illness (D0-D3), including alterations in the metabolic clock associated with pair feeding in vehicle-treated mice from D2, and a leftward shift in RER. Animals were pair-fed twice daily at the beginning of the light (06:00) and dark (18:00) phases to avoid consumption of all food during one timeframe. These alterations did not affect overall health or the muscle parameters investigated.

Despite a clear trajectory towards recovery, the increase in spleen mass persisted in zymosan-treated mice, similar to what has been observed in mice after CLP, despite a restoration of body mass and circulating cytokines returning to baseline levels (Valdés-Ferrer et al., 2013). In the published study, spleen mass only returned to baseline levels 12 weeks after critical illness when CD11b^+^ and Ly-6C^High^ splenic monocytes had returned to baseline. Our observation of a sustained increase in spleen mass could be attributed to the significant inflammatory cell infiltration in the diaphragm muscle 28 days after zymosan-induced critical illness, but this needs to be confirmed in future studies.

The recovery from critical illness was also associated with the restoration of muscle mass and function after the initial wasting and weakness. Atrophy of hindlimb muscles was evident by D7, except in the soleus muscle, which was protected. The TA muscle, comprising a mixture of oxidative and glycolytic fibres, showed atrophy of type IIB fibres that was restored by D14. Glycolytic muscle fibres tend to be larger (in mice) with a higher force-generating capacity than slow, oxidative muscle fibres, but they are less capable of managing oxidative stress (Lloyd et al., 2023). Type IIB muscle fibres can also potentiate reactive oxygen species generation and oxidative stress (Anderson and Neufer, 2006). This is supported by the soleus muscle being protected from zymosan-induced wasting, despite a significant increase in monocyte infiltration.

Restoration of TA muscle mass and function after zymosan administration confirmed previous findings in rats (Hill et al., 2015). In a study of more severe zymosan-induced critical illness, muscle function only partially recovered at D11 compared to D6 (Minnaard et al., 2005). In the present study, atrophy of the TA muscles at D4 and D7 after zymosan administration was associated with a reduction in peak tetanic force and a reduction in specific force, indicating the impaired muscle function was associated with factors other than just a loss of muscle mass. As critically ill patients can experience neuromuscular deficits (Friedrich et al., 2015), further investigation is required to determine whether such deficits (including impaired neurotransmission) contribute to this dysfunction in mice. Neuromuscular abnormalities such as axonal degeneration have been reported in gastrocnemius muscles of mice 5 days after CLP surgery (Goossens et al., 2021), although this study identified skeletal muscle and not peripheral nerves as the main contributor to sepsis-induced muscle weakness.

The restoration of muscle function by D14 showed that functional recovery was possible after critical illness in this model. Our functional assessments of an isolated (TA) muscle rather than of a muscle group, such as dorsi/plantar flexors (Hill et al., 2015; Minnaard et al., 2005; Rooyackers et al., 1997), is advantageous as it allows for targeted analyses of how critical illness affects specific fibres and their contributions to overall muscle function.

Although our zymosan model in mice facilitated assessment of functional recovery, it should be noted that the mice were relatively young and healthy (15-16 weeks of age) with no evidence of prior muscle dysfunction. Longitudinal studies in much older mice (16 months of age) showed persistent muscle dysfunction after CS-induced sepsis (Kingren et al., 2024; Owen et al., 2019), which may be attributed to compromised muscle health with ageing. This is an important consideration as many patients admitted to the ICU are older adults. Studies comparing responses to zymosan in young and aged mice would provide further insight into the mechanisms of wasting and recovery with critical illness.

Inflammatory cell infiltration was evident in the diaphragm over the 28-day period compared with that in the hindlimb muscles, where monocytes were cleared before muscle mass was restored. In critically ill patients, diaphragm atrophy is associated with sepsis (Lu et al., 2019) and mechanical ventilation (Daniel Martin et al., 2013), with isolated muscle fibres obtained from diaphragm biopsies of mechanically ventilated patients exhibiting significant functional deficits (van den Berg et al., 2024). In animal models of sepsis, impaired specific force of diaphragm muscle strips *ex vivo* is apparent as early as 18 h (Jiang et al., 2015) and 24 h (Supinski et al., 2020) after CLP surgery. Delayed wasting of the diaphragm muscle has been observed in other models associated with long-term inflammation, such as cancer, with wasting evident after immune cell infiltration (Neyroud et al., 2025). In a mouse model of pancreatic cancer induced by orthotopic murine pancreatic tumours, leukocyte infiltration occurred at D8, followed by muscle fibre atrophy, with preferential wasting of type IIB/X fibres at D12 (Neyroud et al., 2025).

The greater burden on the diaphragm than on hindlimb muscles after zymosan administration could be attributed to the proximity of the insult within the peritoneal cavity. Adhesion of microgranulomas appeared to worsen over time, and those on the abdominal side of the diaphragm could potentially limit nutrient delivery to muscle fibres, resulting in atrophy. Whether these microgranulomas contain undegraded particles of zymosan remains to be determined, but microgranulomas have been reported in the peritoneal cavity after zymosan administration (Goris et al., 1986; Rooyackers et al., 1994). Further investigation into the origins of these diaphragm muscle deficits is warranted based on their implications for long-term recovery.

Respiratory dysfunction was evident at D1 after zymosan administration, consistent with reductions in VO_2_. Similar respiratory deficits, based on WBP, were observed in septic mice 24 h after CLP with recovery at 48 h (Iskander et al., 2013). We found no respiratory deficits when the mice were being assessed in the metabolic cages and no deficits in overall respiratory function (at rest) despite the aberrations in diaphragm structure. It is possible that functional deficits in the diaphragm (at rest) might not be detected with WBP, especially as compensation from external respiratory muscles is possible. WBP responses to a hypercapnic or exercise challenge can identify respiratory dysfunction (Getsy et al., 2023; Burns et al., 2017), and these should be considered in future studies. The severe diaphragm dysfunction in the *mdx* and other mouse models of Duchenne muscular dystrophy has been detected using WBP, with these deficits being associated with significant collagen infiltration (Huang et al., 2011). The diaphragm pathology in these dystrophic mice is more severe than that in critically ill mice after zymosan administration. Regardless, the differential responses of diaphragm and hindlimb muscles after zymosan treatment is interesting and further studies are warranted.

This study investigated the time course of muscle functional recovery after zymosan-induced critical illness in mice. Zymosan administration induced a critical illness phenotype, with initial muscle atrophy and functional impairments of muscles, but recovery of muscle function after 14 days. The findings support the hypothesis that recovery from muscle wasting and weakness is possible after critical illness, and that the zymosan model in mice provides a suitable platform for mechanistic investigations towards therapeutic advances for ICU-AW.

## MATERIALS AND METHODS

### Zymosan preparation

Zymosan A extracted from *Saccharomyces cerevisiae* (zymosan; Z4250, CAS no. 58856-93-2, Sigma-Aldrich) was sterilised (120 Gy) using a cobalt 60 γ irradiator. A suspension was created (25 mg/ml) through homogenisation in sterile light liquid paraffin (OVOIL, VitroLife, Gothenburg, Sweden) using an OMNI 1000 homogeniser (OMNI International, Kennesaw, GA, USA) and modified based on procedures described previously (Rooyackers et al., 1994; Minnaard et al., 2005).

### Animals

Mice (15- to 16-week-old C57BL/6J males) were sourced from Ozgene (Perth, Australia) and housed in the Biomedical Sciences Animal Facility (The University of Melbourne) under a 12 h:12 h light-dark cycle at a temperature of 22±1°C, a humidity of 40-70% and *ad libitum* access to water for the study duration. Mice were allocated to one of three experimental groups: control, vehicle or zymosan (*n*=7-10 mice/group/timepoint). Zymosan-treated mice received an intraperitoneal injection of zymosan (30 mg/100 g), suspended in liquid paraffin (25 mg/ml), with vehicle-treated mice receiving an equivalent volume of liquid paraffin. All vehicle-treated mice received the same amount of standard chow (22% crude protein, 9% crude fat, 3.2% crude fibre, 25% starch; Barastoc, Melbourne, Australia) as their zymosan-treated (critically ill) counterparts. Zymosan-treated and control mice received *ad libitum* access to standard chow for the study duration. Mice were monitored daily for clinical signs and symptoms mimicking critical illness (including piloerection, lethargy, anorexia and laboured respiration) with terminal collection of tissues at D4, D7, D14 and D28 after mice had been allocated to their groups (control, vehicle and zymosan).

At experimental endpoint, mice were anaesthetised deeply with sodium pentobarbitone (60 mg/kg i.p.; Sigma-Aldrich) and euthanised via cardiac excision. All muscles and organs were rapidly excised and either snap frozen in liquid nitrogen or embedded in optimal cutting temperature embedding compound (OCT; Sakura Finetek, Torrance, CA, USA) and frozen in cooling isopentane (Sigma-Aldrich). Tibia length was measured using digital callipers for normalisation purposes. All tissue was stored at −80°C for subsequent analyses.

### Metabolic phenotyping and assessment of body composition

In a separate cohort of mice (*n*=4-5 per group), comprehensive metabolic phenotyping was undertaken using the Promethion 16 metabolic cage system (Sable Systems International, Las Vegas, NV, USA) in accordance with the manufacturer's instructions. Briefly, after acclimation, continuous recordings were made for 6 days with data extracted every 5 min using MetaScreen (Sable Systems International) and ExpeData (Sable Systems International). Measures of VO_2_ and VCO_2_ were obtained via a gas analyser. Non-invasive measures of energy expenditure and RER were calculated via indirect gas exchange calorimetry. Energy expenditure was calculated using the Weir equation [(3.94×VO_2_)+(1.1×VCO_2_)] (Weir, 1949). For activity measures, mouse location and movement were recorded second by second using *xy*z beam-break analyses. ‘AllMetres’ represents the sum of all distances travelled.

Measurements of whole-body fat mass, lean mass and free body fluid were obtained via time domain nuclear magnetic resonance imaging (Bruker LF50 MiniSpec, Bruker, Billerica, MA, USA) in accordance with the manufacturer's instructions on D−1, D4, D7 and D13.

### Whole-body plethysmography (WBP)

Measurements of respiratory function in conscious, unrestrained mice were obtained using WBP (Buxco, Data Sciences International, Saint Paul, MN, USA) as described previously (Verzele et al., 2024). The system consisted of a sealed Plexiglas chamber that was continually filled with fresh room air (not pressurized) and the airflow variability in the chamber (associated with breathing movements) was monitored with sensitive flow transducers. Animals were placed in the plethysmography chamber and acclimated for 20 min, before basal respiration was measured continuously for an additional 40 min. Measurements were obtained at D−1, D1, D4, D7, D14, D21 and D28. The data reported relate to the 20 min of recording after the acclimation period when the mice were under resting conditions (with limited movement and grooming).

### Assessments of skeletal muscle function

Assessments of TA muscle function were performed *in situ* as described previously (Swiderski et al., 2024). Briefly, at experimental endpoint, mice were anaesthetised with sodium pentobarbitone (60 mg/kg). An incision was made in the skin of the right hindlimb and the TA muscle and distal tendon were exposed. The distal tendon was secured to a force transducer (model 305B-LR dual-mode lever system, Aurora Scientific, Aurora, ON, Canada) and the sciatic nerve of the right hindlimb was exposed for stimulation. The mouse was placed laterally onto a custom-built platform (water-jacketed Plexiglas) with the knee immobilised and the lower-right hindlimb bathed in warmed mineral oil (37°C; Sigma-Aldrich).

Muscle length was adjusted to achieve the optimal length (L_o_) to produce maximum isometric twitch force (P_t_) and peak tetanic force (P_o_) determined from the plateau of the frequency-force relationship (stimulating from 10-300 Hz, with a pulse duration of 350 ms and 2 min rest between tetanic contractions). The TA muscle was then subjected to an intermittent stimulation fatigue protocol by eliciting maximum tetanic contractions every 4 s for 4 min. P_o_ was also determined after 5 and 10 min of rest (i.e. no stimulation) at the end of the fatigue protocol to determine the ability of the muscle to recover maximum force-producing capacity.

### Skeletal muscle histology

Transverse sections of tissue (8 μm) were cut at −20°C using a cryostat (Minux FS800A, RWD Life Science, San Diego, CA, USA). All slides were stored at −80°C and staining protocols were conducted at room temperature unless otherwise stated.

Assessments of inflammatory cell infiltration (CD45 and CD68) were performed via immunofluorescence as described previously (Saunders et al., 2025), except for the use of relevant primary and secondary antibody cocktails listed in Tables S2 and S3, respectively.

General assessments of muscle fibre typing were performed as described previously (Swiderski et al., 2024) with the following exceptions. After fixing, sections were incubated in primary antibody cocktail [in PBS with 0.05% Tween 20 (PBST), Sigma-Aldrich; Table S4] in a humidity chamber for 1.5 h. Slides were washed in PBS and incubated for 1.5 h in the relevant secondary antibody (in PBST; Table S5) in a humidity chamber. Slides were washed in PBS and mounted with Mowiol 4-88 mounting medium for immunofluorescence (Sigma-Aldrich) and a coverslip added before being left to dry in the dark at room temperature overnight. Slides were stored in the dark at 4°C prior to imaging. Muscle fibres that were myosin heavy chain type I, IIA and IIB negative were determined to be myosin heavy chain type IIX fibres.

To test for cellular integrity, H&E staining was performed as described previously (Swiderski et al., 2019).

All sections were imaged using the Axioscan 7 slide scanner using ZEN 3.5 Slidescan (Blue), ZEN 3.5 Desk and ZEN Connect software (Zeiss, Oberkochen, Germany) at 10× magnification and 2×2 binning, with 16-bit images analysed using ImageJ (FIJI; National Institutes of Health). MinFeret diameter was used to assess myofibre diameter from laminin staining. Mean muscle fibre CSA was determined from laminin staining (Swiderski et al., 2024), and analysis of IgG, CD45 and CD68 staining intensity (percentage of area) was performed in accordance with IgG analyses described previously (Swiderski et al., 2026) using ImageJ.

### Statistical analyses

All values are expressed as mean±s.e.m. unless stated otherwise. All muscle and organ masses were normalised to tibia length as this was unaltered between groups. All graphs and statistical analyses were generated using GraphPad Prism v10.0 (GraphPad Software, San Diego, CA, USA). Comparisons involving more than two groups were analysed using a one-way ANOVA with Tukey's multiple comparisons test. Comparisons between treatment groups over time were analysed using a two-way ANOVA with Tukey's multiple comparisons test, or a mixed-effect analysis with Tukey's multiple comparisons test. Comparisons between two groups were analysed using a Student's two-tailed unpaired *t*-test. Post-hoc analyses were only performed when the overall ANOVA or mixed-effect test was determined to be significant (*P*<0.05).

### Ethics statement

All experiments were approved by the Animal Ethics Committee of The University of Melbourne and conducted in accordance with the Australian code for the care and use of animals for scientific purposes, as stipulated by the National Health and Medical Research Council (Australia, 2013). All aspects of the study adhered to the ARRIVE guidelines for reporting *in vivo* experiments in animal research.

## Supplementary Material

10.1242/dmm.052712_sup1 Supplementary information
