# Supplementary material for: Evaluating skeletal muscle dysfunction and recovery in a zymosan model of critical illness in mice
Source: Dis Model Mech. 2026 Jun 1;19(5):dmm052712. doi: 10.1242/dmm.052712 (PMC13267770; doi:10.1242/dmm.052712)
Supplement: Supplementary information [file dmm-19-052712-s1.pdf]

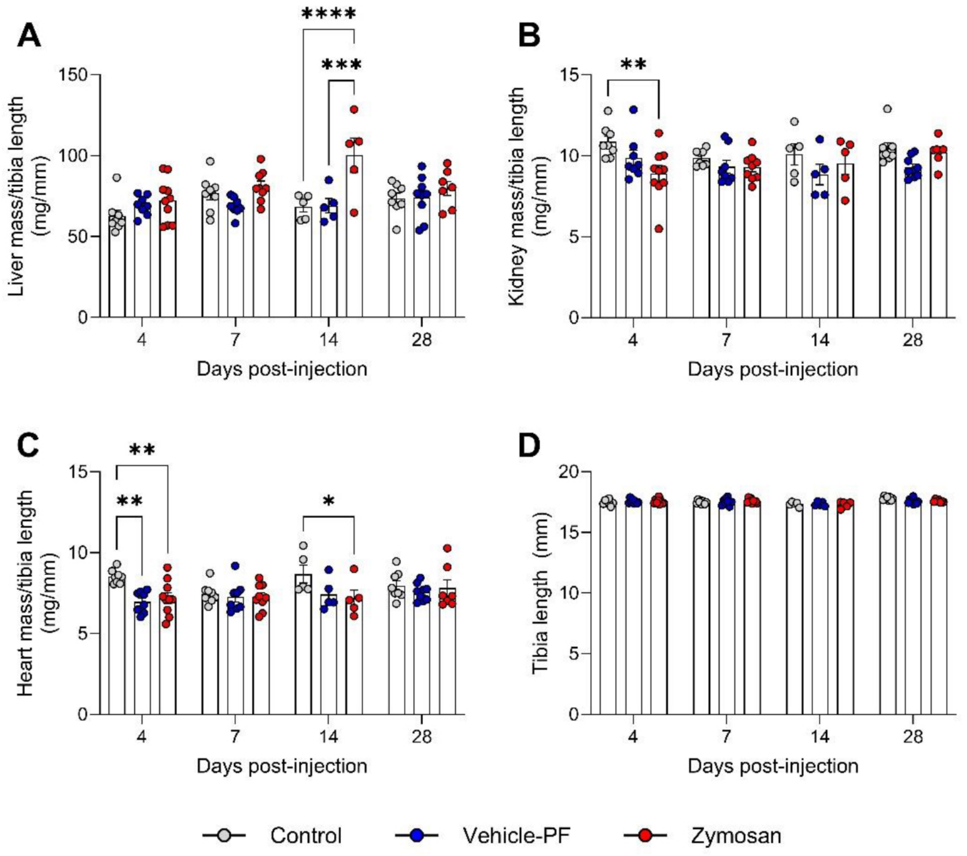

Fig. S1. Zymosan administration increases liver mass after 14 days. C57BL/6J male mice (15-16 wks) were allocated to control, vehicle or zymosan groups, with samples collected at 4, 7, 14 and 28 days after critical illness induction. Liver mass of zymosan treated mice was increased at D14 compared with vehicle treated and control mice (A), with minimal changes in kidney (B) and heart mass (C). Tibia length was used for normalisation purposes as it remained unchanged throughout the study (D). PF: pair-fed. Data are mean ± SEM, n=5-10/group. *P<0.05, **P<0.01, ***P<0.001, ****P<0.0001.

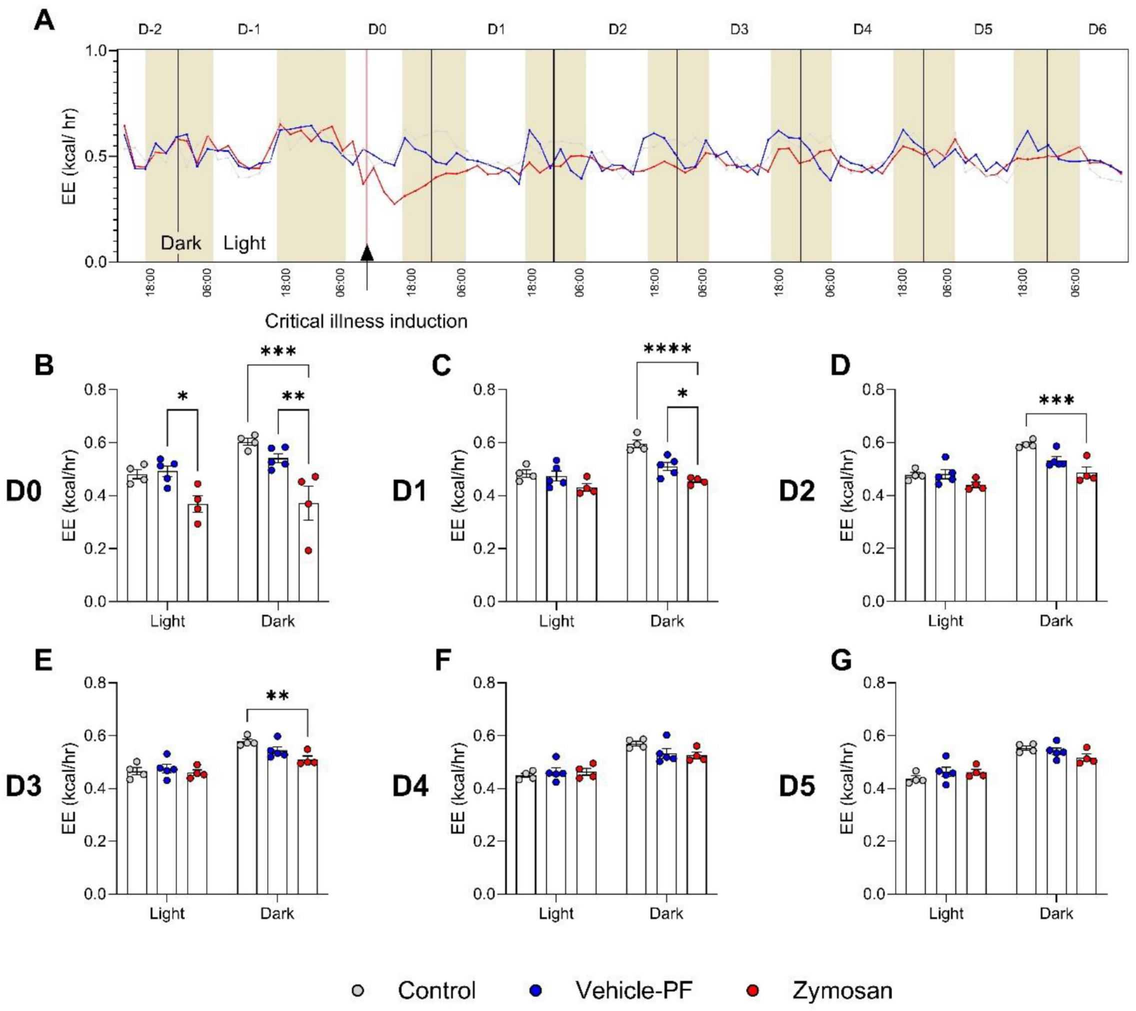

Fig. S2. Assessment of energy expenditure in critically ill mice after zymosan-induced peritonitis. C57BL6/J male mice (15-16 wks) were allocated to either control, vehicle or zymosan groups before undergoing whole-body metabolic phenotyping using the Promethion 16-cage system to assess EE (A). Analysis of EE 24 hrs (D0; B) and at D1 (C), D2 (D), D3 (E), D4 (F) and D5 (G) after the induction of critical illness. For A, data are presented at 2 hr intervals. D: day, EE: energy expenditure, PF: pair-fed. Data are mean ± SEM, n=4-5/group. *P<0.05, **P<0.01, ***P<0.001, ****P<0.0001.

Disease Models & Mechanisms • Supplementary information

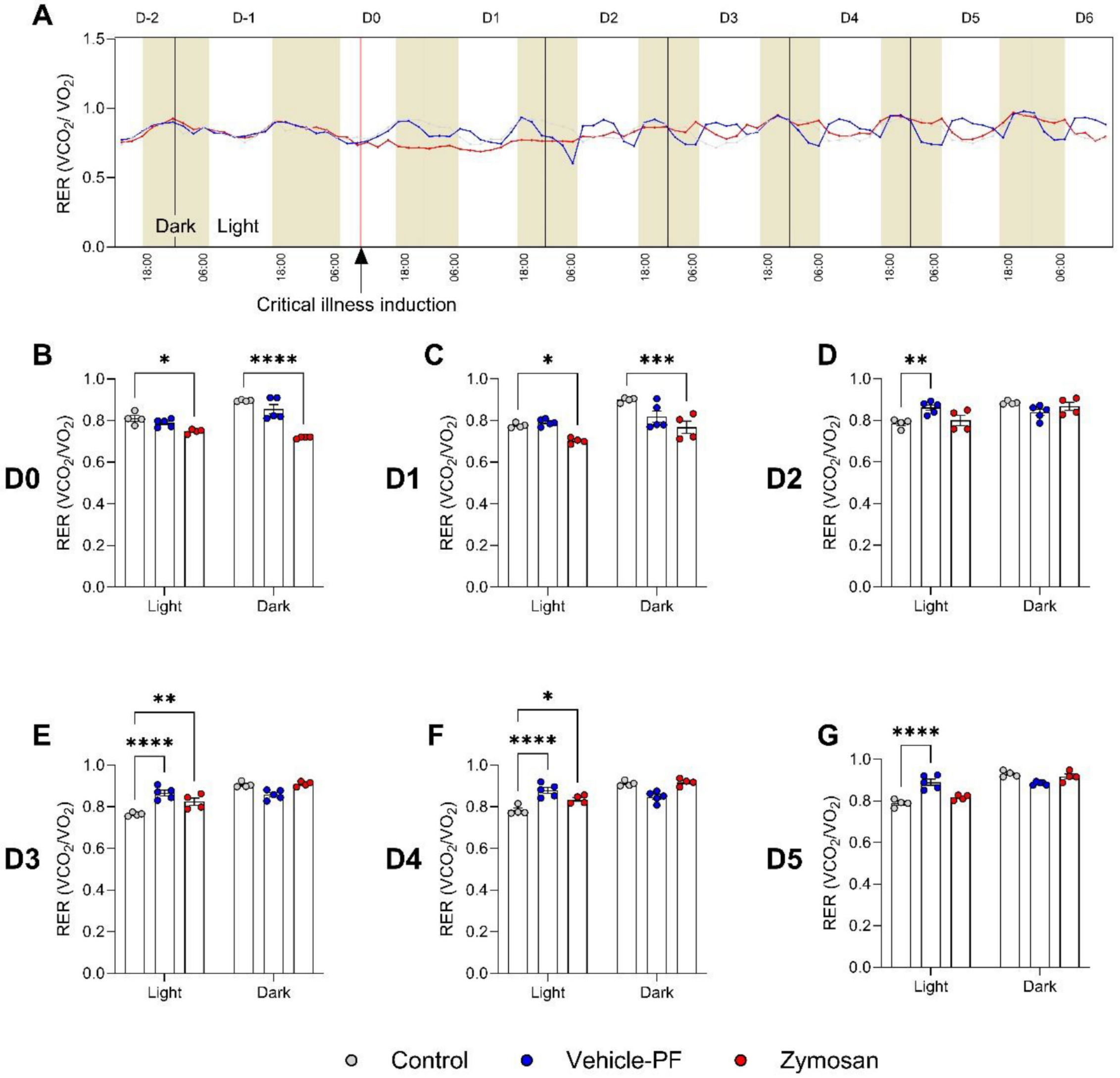

Fig. S3. Assessment of whole-body substrate metabolism in critically ill mice after zymosan-induced peritonitis. C57BL6/J male mice (15-16 wks) were allocated to either control, vehicle or zymosan groups before undergoing whole-body metabolic phenotyping using the Promethion 16-cage system to assess whole-body substrate metabolism (A). Analysis of RER 24 hrs (D0; B) and at D1 (C), D2 (D), D3 (E), D4 (F) and D5 (G) after the induction of critical illness. For A, data are presented at 2 hr intervals. PF: pair-fed, RER: respiratory exchange ratio (VCO_2/VO_2). Data are mean ± SEM, n=4-5/group. *P<0.05, **P<0.01, ***P<0.001, ****P<0.0001.

Disease Models & Mechanisms • Supplementary information

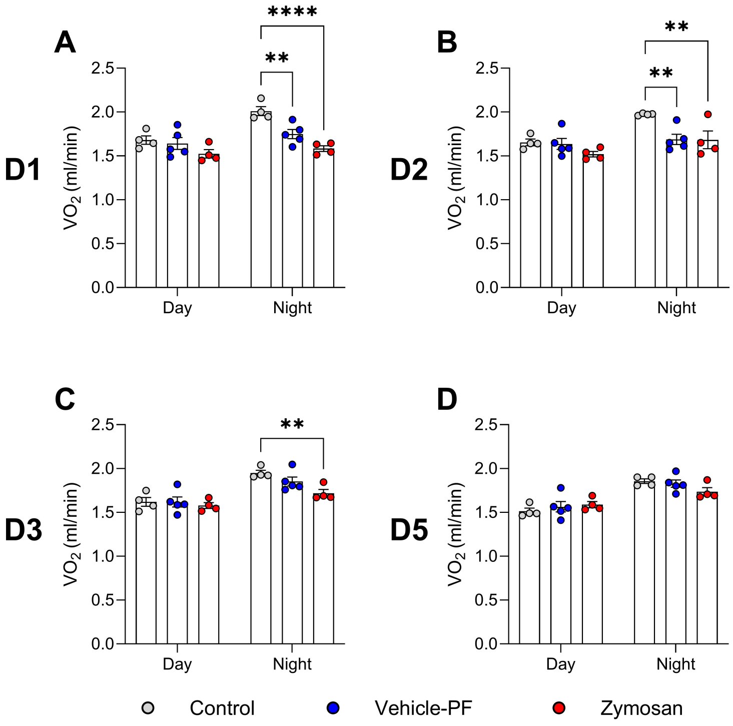

Fig. S4. Assessment of oxygen consumption in critically ill mice after zymosan-induced peritonitis. C57BL6/J male mice (15-16 wks) were allocated to either control, vehicle or zymosan groups before undergoing whole-body metabolic phenotyping using the Promethion 16-cage system. Analysis of VO_2 at D1 (A), D2 (B), D3 (C) and D5 (D) after induction of critical illness. D: day, PF: pair-fed, VO_2: oxygen consumption. Data are mean ± SEM, n=4-5/group. **$P<0.01$, ****$P<0.0001$.

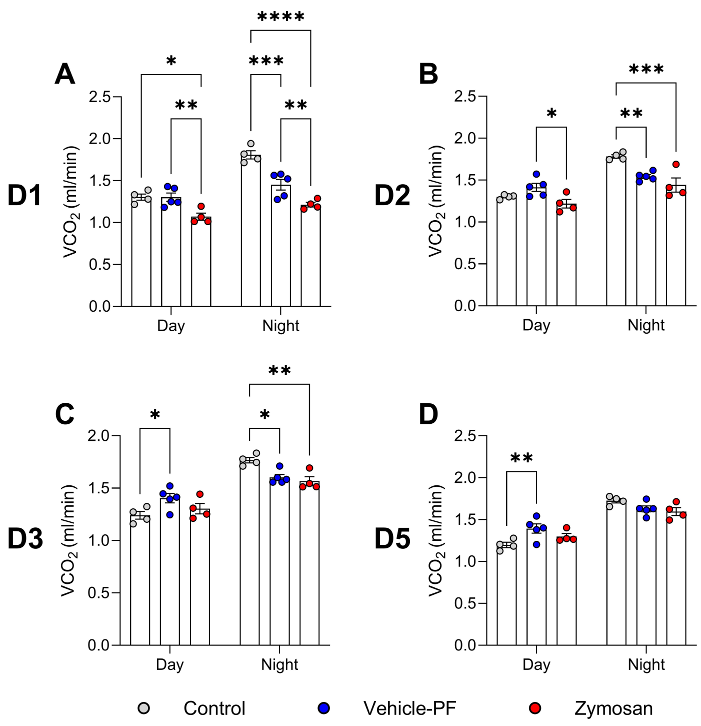

Fig. S5. Assessment of carbon dioxide production in critically ill mice after zymosan administration. C57BL6/J male mice (15-16 wks) were allocated to either control, vehicle or zymosan groups before undergoing whole-body metabolic phenotyping using the Promethion 16-cage system. Analysis of VCO_2 at D1 (A), D2 (B), D3 (C) and D5 (D) after the induction of critical illness. D: day, PF: pair-fed, VCO_2: carbon dioxide production. Data are mean ± SEM, n=4-5/group. *P<0.05, **P<0.01, ***P<0.001, ****P<0.0001.

Disease Models & Mechanisms • Supplementary information

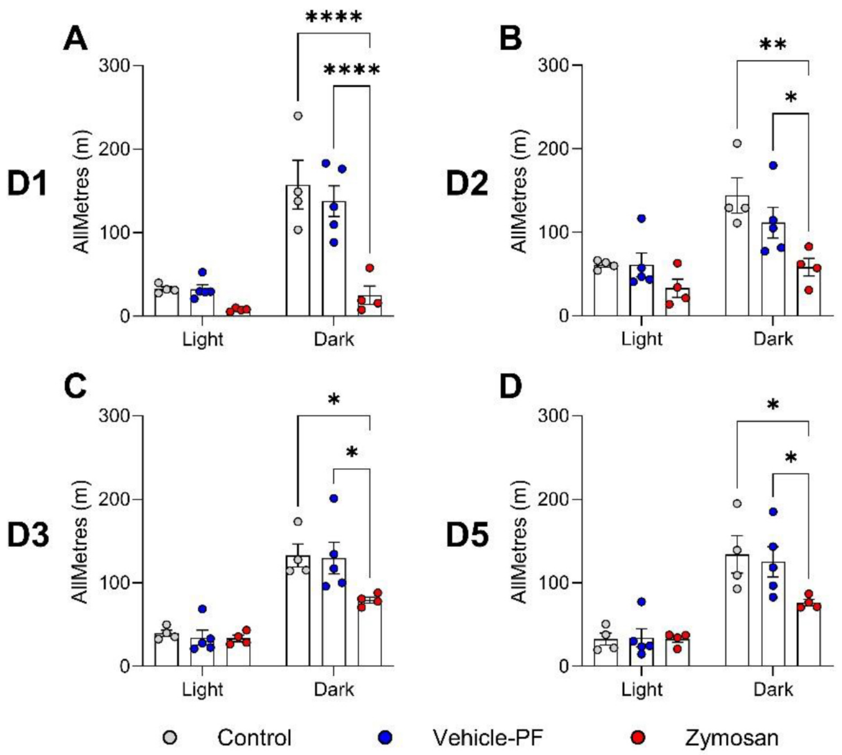

Fig. S6. Assessment of ambulation in critically ill mice after zymosan administration. C57BL6/J male mice (15-16 wks) were allocated to either control, vehicle or zymosan groups before undergoing whole-body metabolic phenotyping using the Promethion 16-cage system. Analysis of ambulation at D1 (A), D2 (B), D3 (C) and D5 (D) after induction of critical illness. D: day, PF: pair-fed. Data are mean ± SEM, n=4-5/group. *P<0.05, **P<0.01, ****P<0.0001.

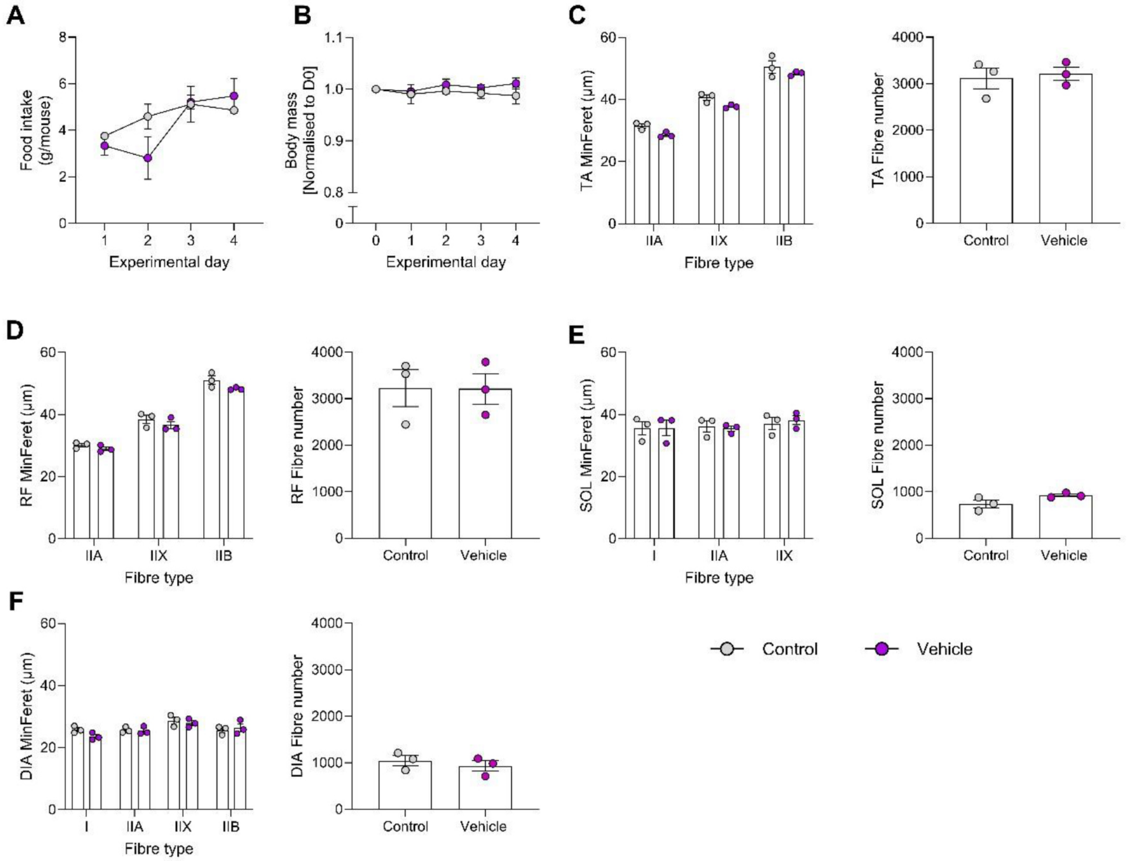

Fig. S7. Vehicle administration does not cause muscle atrophy in mice after 4 days. *Ad libitum* feeding of vehicle treated mice revealed the muscle phenotype in pair-fed vehicle treated mice was attributed entirely to caloric restriction. No changes were evident in food intake (A) and body mass (B) between groups. There were no changes in muscle fibre diameter (MinFeret) or fibre number, respectively, in the TA (C), RF (D), SOL (E) and DIA (F) muscles. TA: tibialis anterior, RF: rectus femoris, SOL: soleus, DIA: diaphragm. Data are mean ± SEM, n=3/group.

Disease Models & Mechanisms • Supplementary information

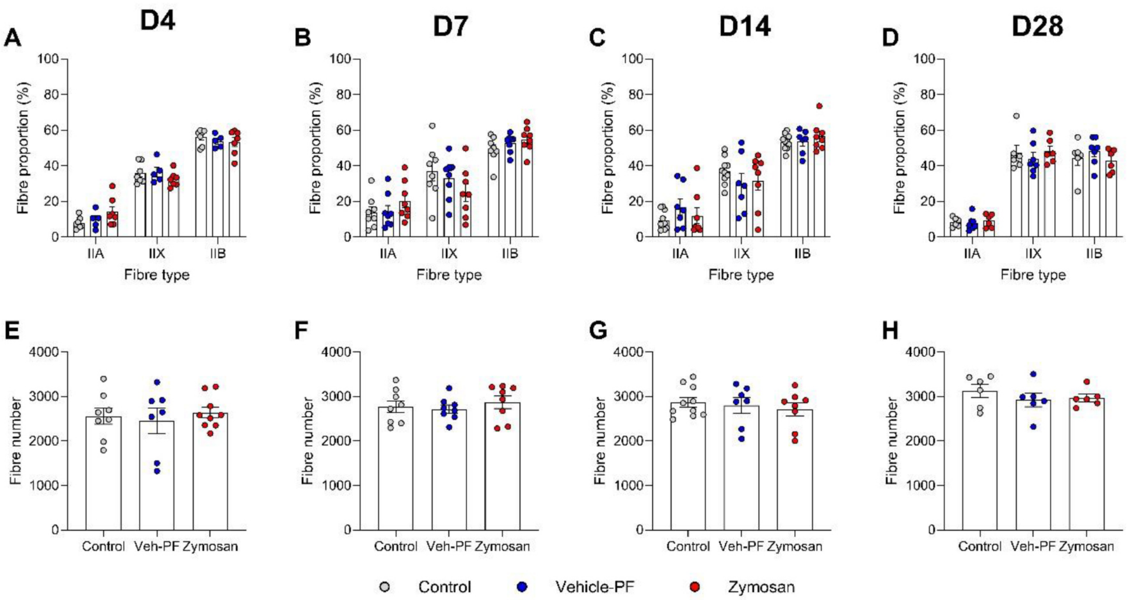

Fig. S8. Analysis of fibre proportions and fibre number in tibialis anterior (TA) muscles of mice after zymosan adminsitration. C57BL/6J male mice (15-16 wks) were allocated to control, vehicle or zymosan groups, with samples collected at 4, 7, 14 and 28 days after critical illness induction. Fibre proportions at D4 (A), D7 (B), D14 (C) and D28 (D) after critical illness. Overall fibre number remained unaltered at D4 (E), D7 (F), D14 (G) and D28 (H) after critical illness. PF: pair-fed, Veh-PF: vehicle-pair fed. Data are mean ± SEM, n=6-10/group.

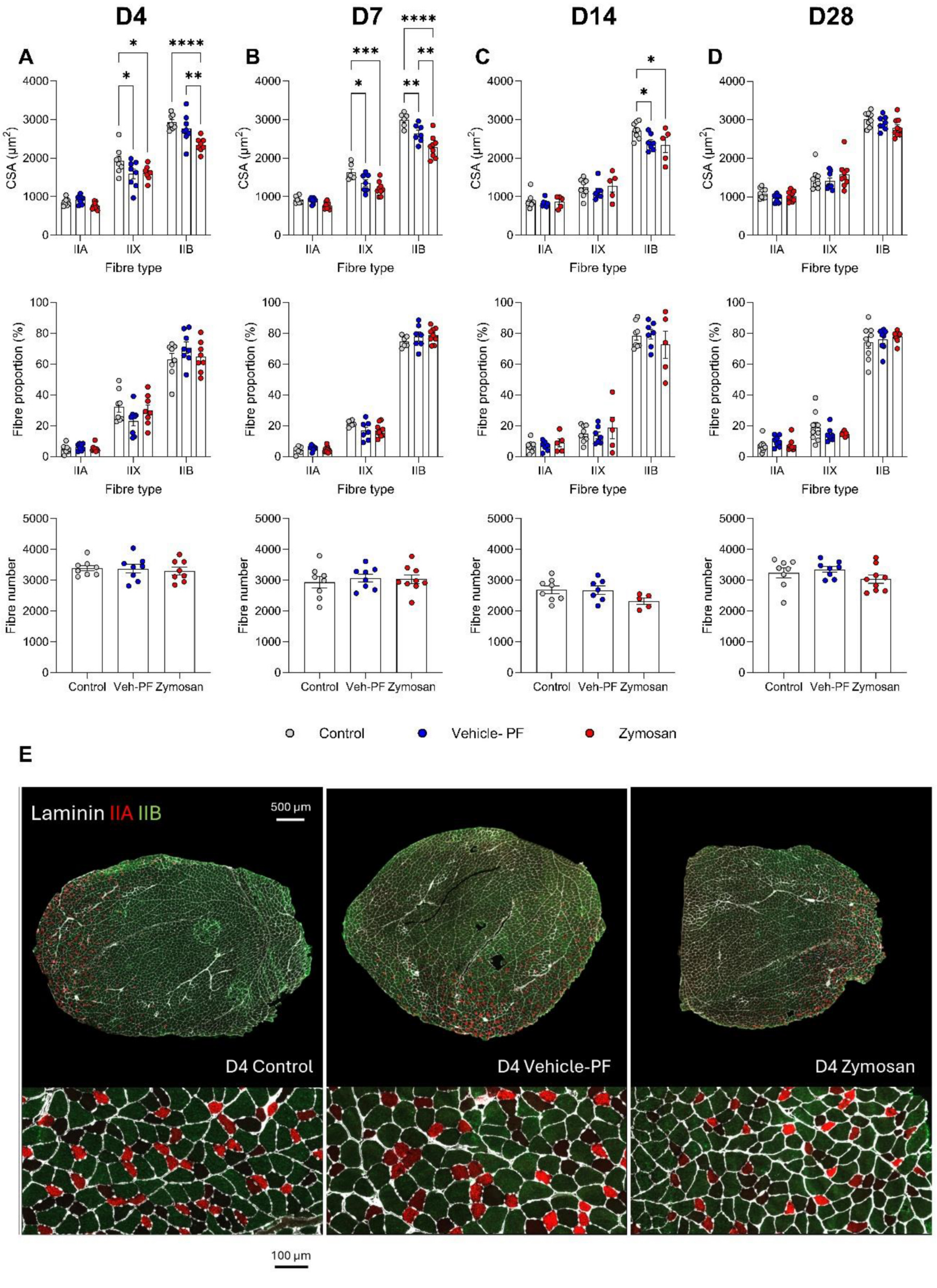
D4
D7
D14
D28
A
B
C
D
CSA (μm²)
Fibre type
IIA
IIX
IIB
Fibre proportion (%)
Fibre number
Control
Veh-PF
Zymosan
Vehicle- PF
E
Laminin IIA IIB
500 μm
D4 Control
D4 Vehicle-PF
D4 Zymosan
100 μm

Fig. S9. Analysis of fibre size (CSA) and fibre types within rectus femoris muscles of mice after zymosan-induced critical illness. C57BL/6J male mice (15-16 wks) were allocated to control, vehicle or zymosan groups, with samples collected at 4, 7, 14 and 28 days after critical illness induction. CSA by fibre type (top panel), fibre proportion (middle panel) and overall fibre number (bottom panel) assessed at D4 (A), D7 (B), D14 (C), and D28 (D), respectively. Representative images for D4 (E). PF: pair-fed, Veh-PF: vehicle-pair fed, CSA: cross-sectional area. Data are mean ± SEM, n=6-10/group. *P<0.05, **P<0.01, ***P<0.001, ****P<0.0001.

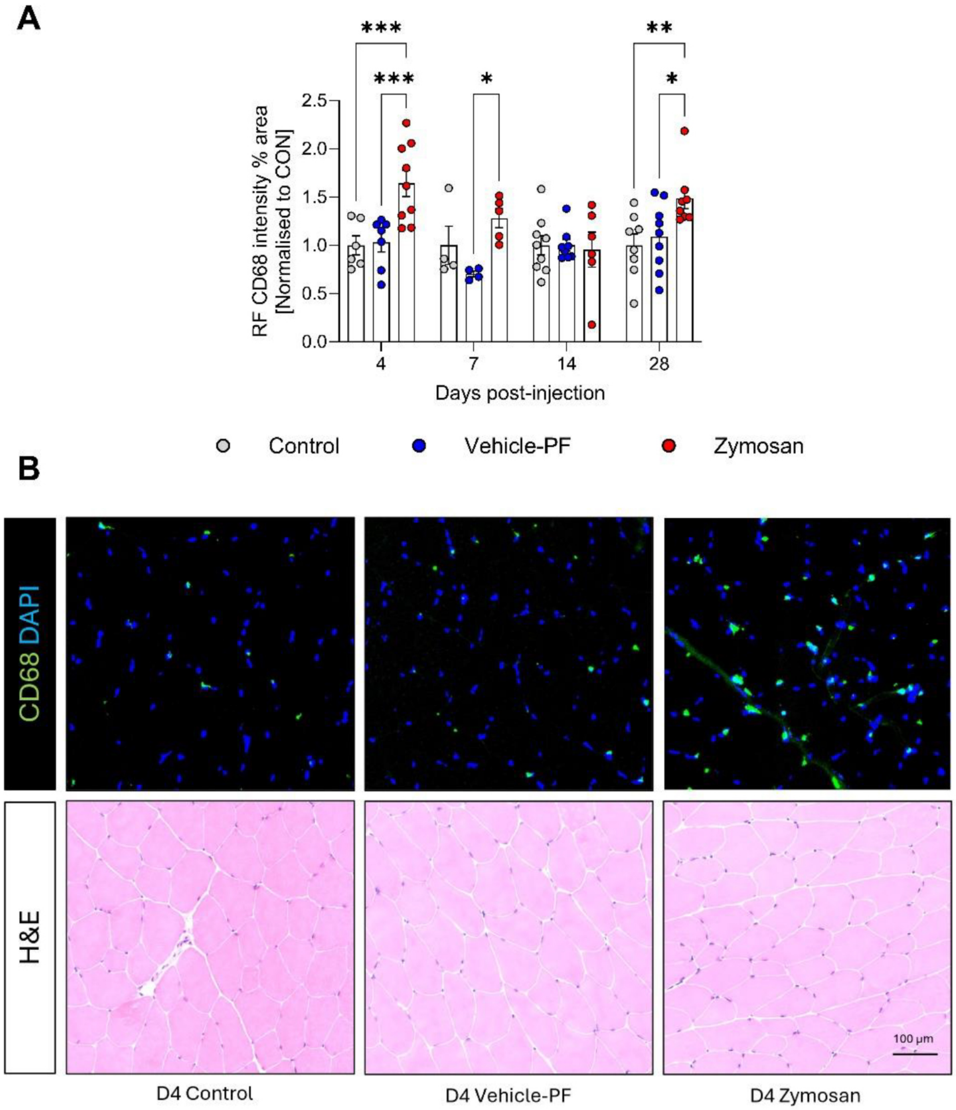

Fig. S10. Monocyte infiltration in rectus femoris muscles in mice after zymosan-induced critical illness. CD68 positive cells were analysed by immunofluorescence at 4, 7, 14 and 28 days after zymosan administration (A), with representative images of $CD68^{+}$ cells (green) and nuclei (DAPI; blue) and H&E-staining revealing no structural damage at D4 (B). H&E: Haematoxylin and eosin staining, CON: control, PF: pair-fed. Data are mean ± SEM, n=4-10/group. **P<0.01, **P<0.01, ***P<0.001.

Disease Models & Mechanisms • Supplementary information

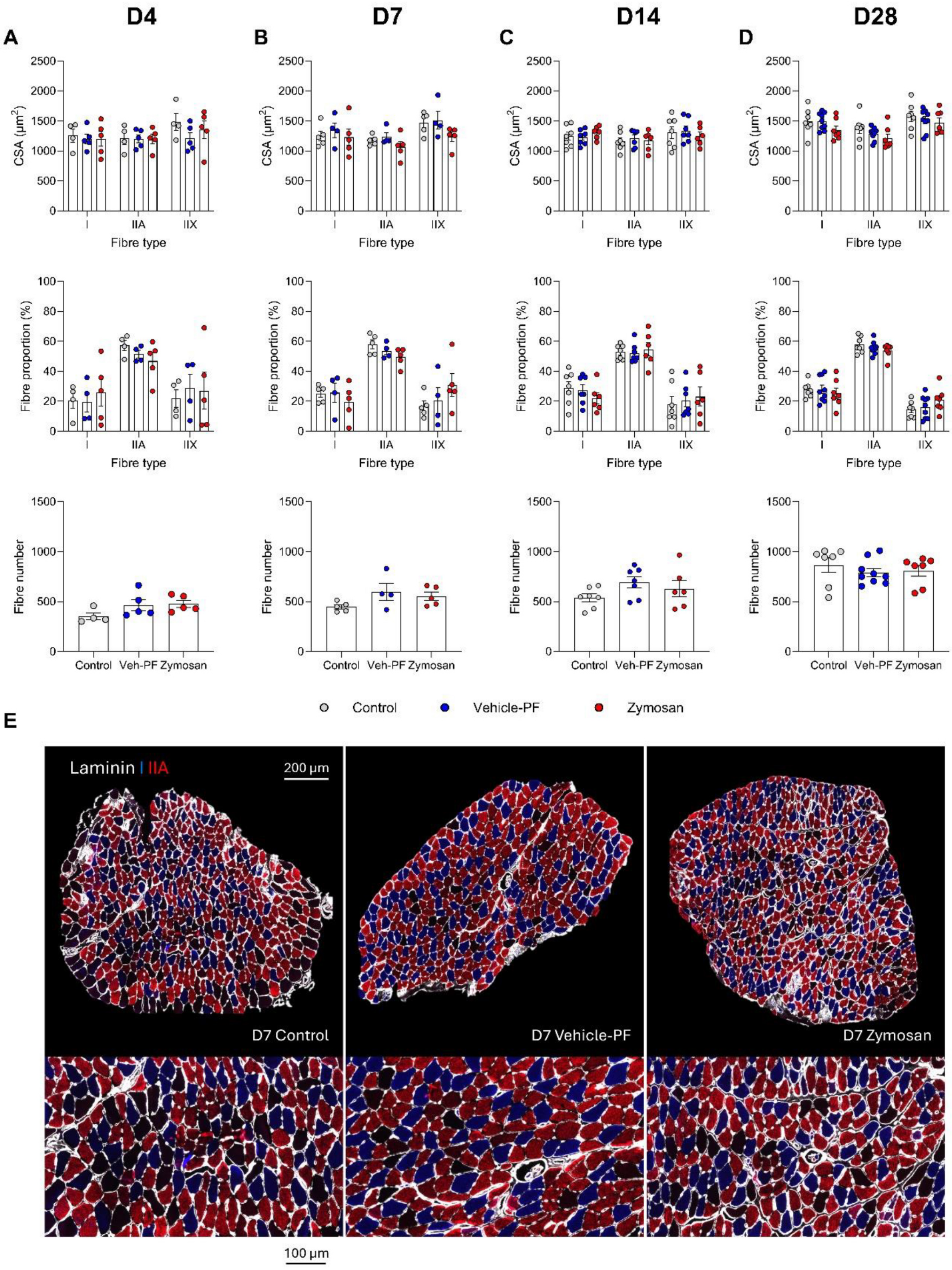

Disease Models & Mechanisms • Supplementary information

Fig. S11. Analysis of fibre size (CSA) and fibre types within the soleus muscles of mice after zymosan-induced critical illness. C57BL/6J male mice (15-16 wks) were allocated to control, vehicle or zymosan groups, with samples collected at 4, 7, 14 and 28 days after critical illness induction. CSA by fibre type (top panel), fibre proportion (middle panel) and overall fibre number (bottom panel) assessed at D4 (A), D7 (B), D14 (C), and D28 (D), respectively. Representative images for D7 (E). PF: pair-fed, Veh-PF: vehicle-pair fed, CSA: cross-sectional area. Data are mean ± SEM, n=6-10/group.

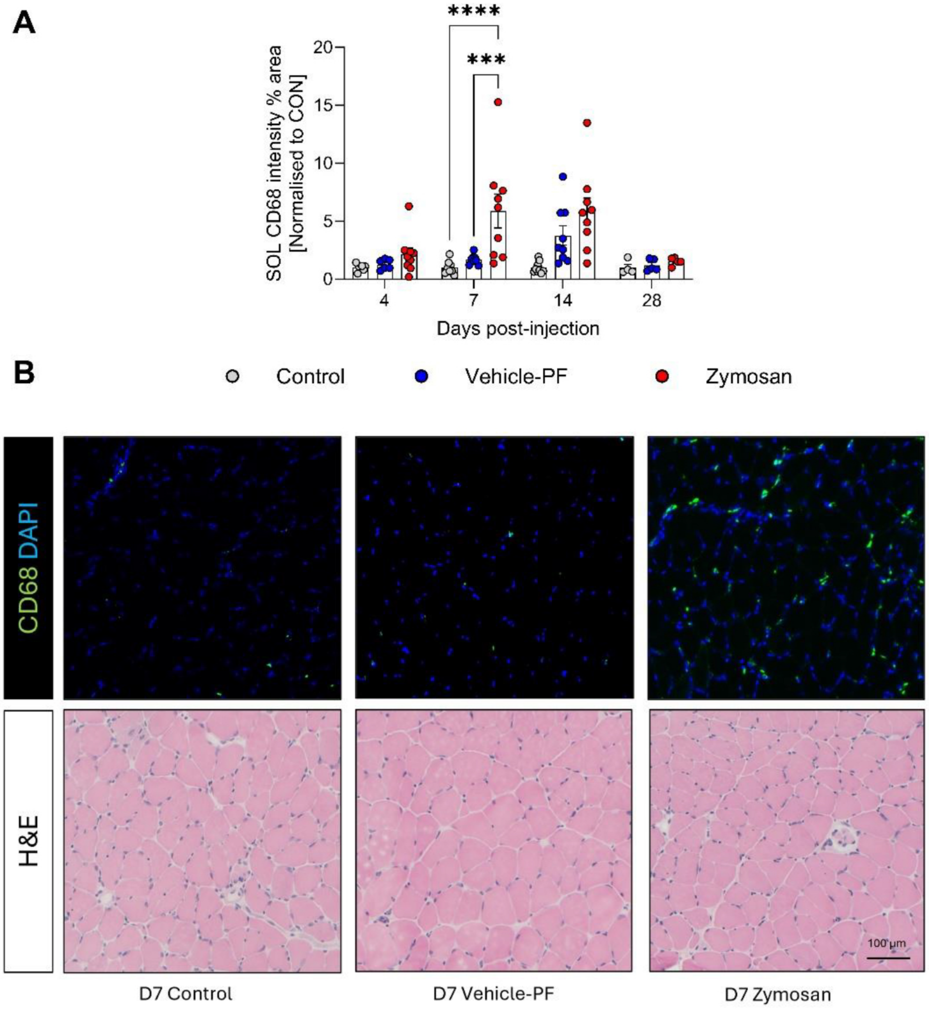

Fig. S12. Monocyte infiltration in soleus muscles of mice after zymosan-induced critical illness. CD68 positive cells were analysed by immunofluorescence at 4, 7, 14 and 28 days after zymosan administration (A), with representative images of $CD68^+$ cells (green) and nuclei (DAPI; blue) and H&E-staining revealing no structural damage at D7 (B). H&E: Haematoxylin and eosin staining, PF: pair-fed. Data are mean ± SEM, n=4-9/group. ***P<0.001, ****P<0.0001.

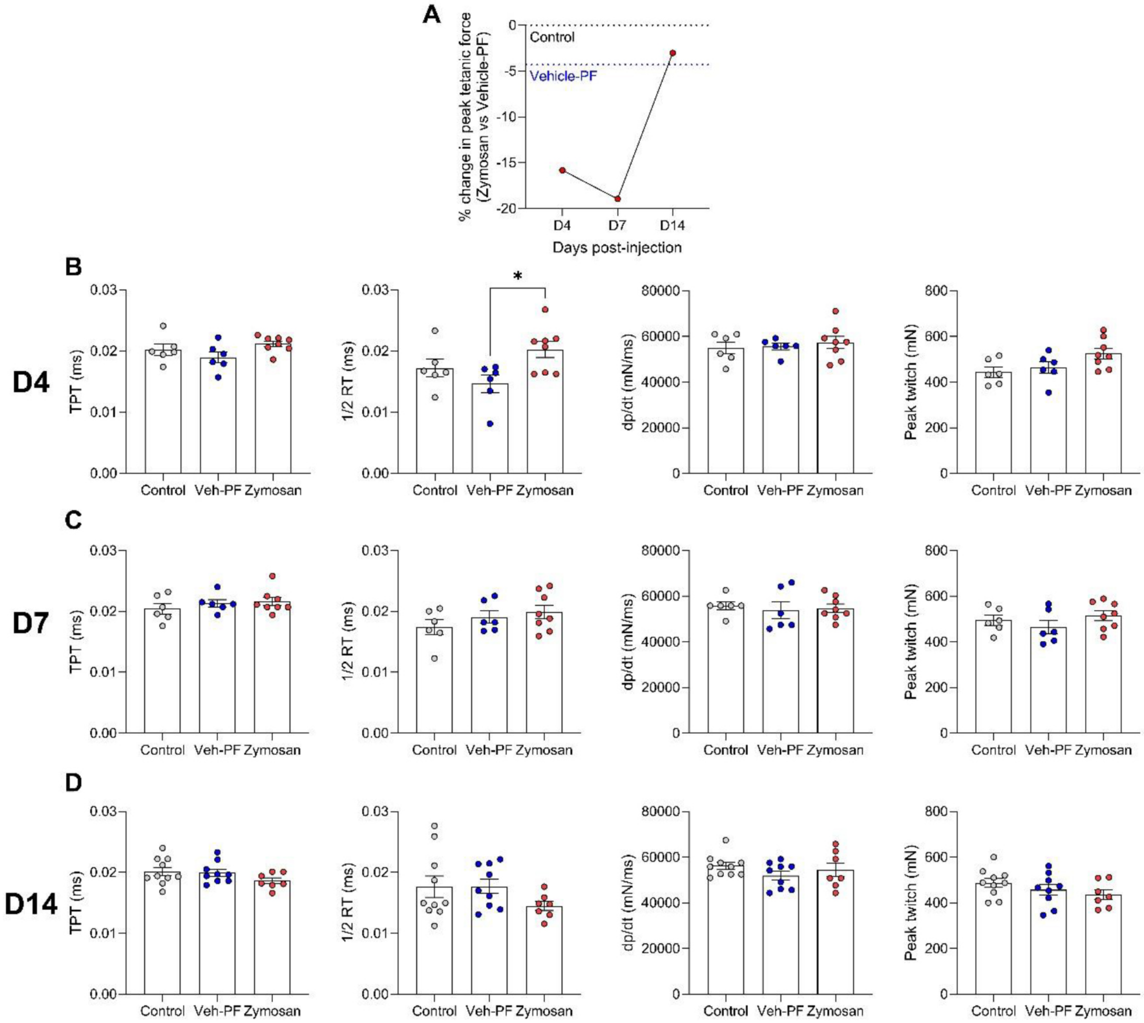

Fig. S13. Contractile properties of TA muscles of mice after zymosan-induced critical illness. Muscle function was assessed at D4, D7 and D14 after zymosan administration. Change in peak force demonstrates functional deficits with subsequent recovery relative to vehicle treated mice (A). Twitch force characteristics were assessed at D4 (B), D7 (C) and D14 (D) with time-to-peak twitch (TPT; left panel), half relaxation time (½ RT; middle left panel), rate of twitch force development (dp/dt; middle right panel) and peak twitch examined (right panel). PF: pair- fed, Veh-PF: vehicle-pair fed. Data are mean ± SEM, n=6-10/group. *P<0.05.

Disease Models & Mechanisms • Supplementary information

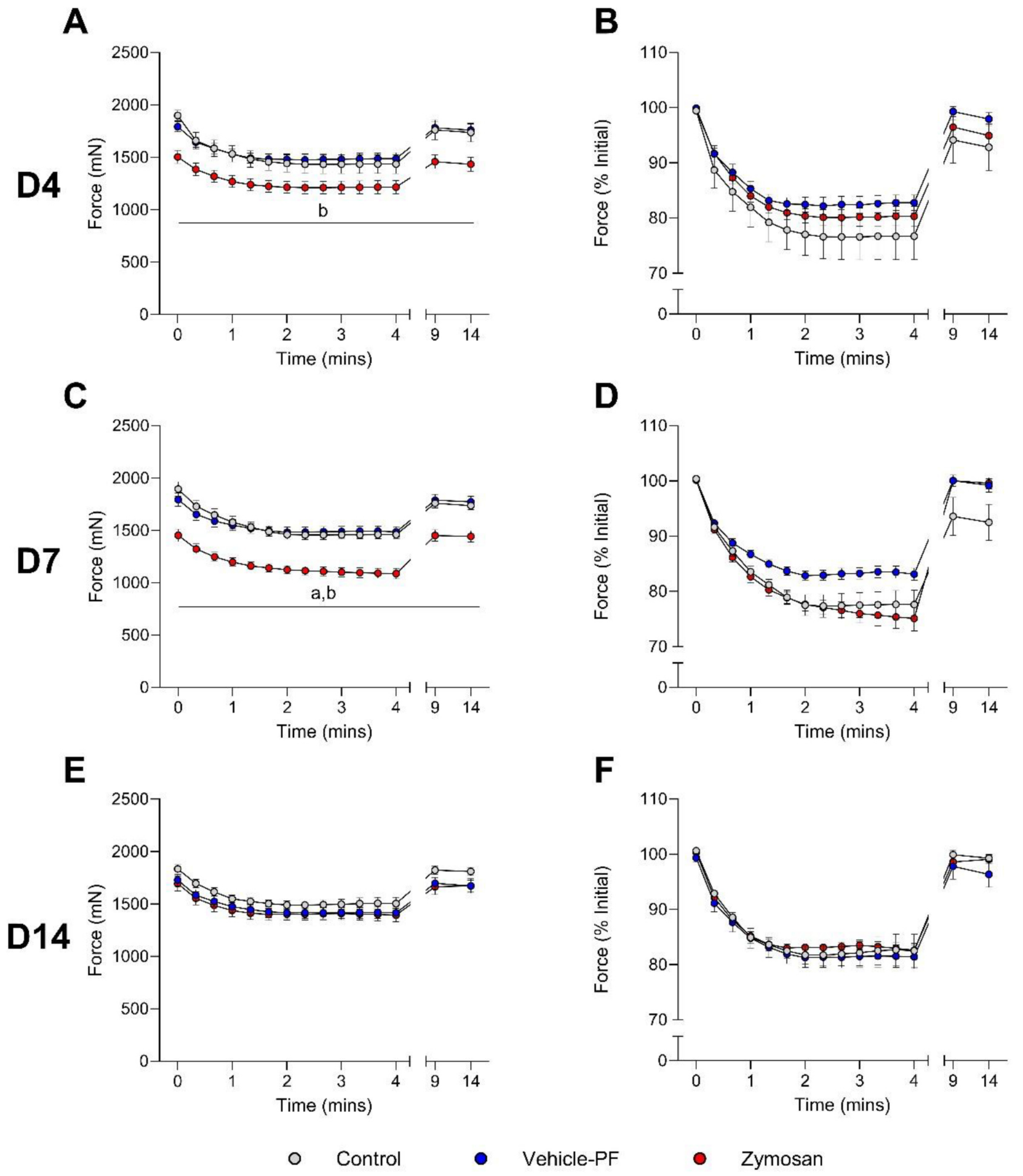

Fig. S14. Skeletal muscle fatigue in TA muscles after zymosan-induced critical illness in mice, assessed *in situ* at D4 (A-B), D7 (C-D), and D14 after zymosan administration (E-F). Muscles were stimulated to produce maximum tetanic force every 4 secs for 4 mins, with peak recovery force also assessed after 5 min and 10 min of rest (i.e., no stimulation). PF: pair- fed. Data are mean ± SEM, n=6-10/group. [a] $P<0.05$ zymosan vs. vehicle, [b] $P<0.05$ zymosan vs. control.

Disease Models & Mechanisms • Supplementary information

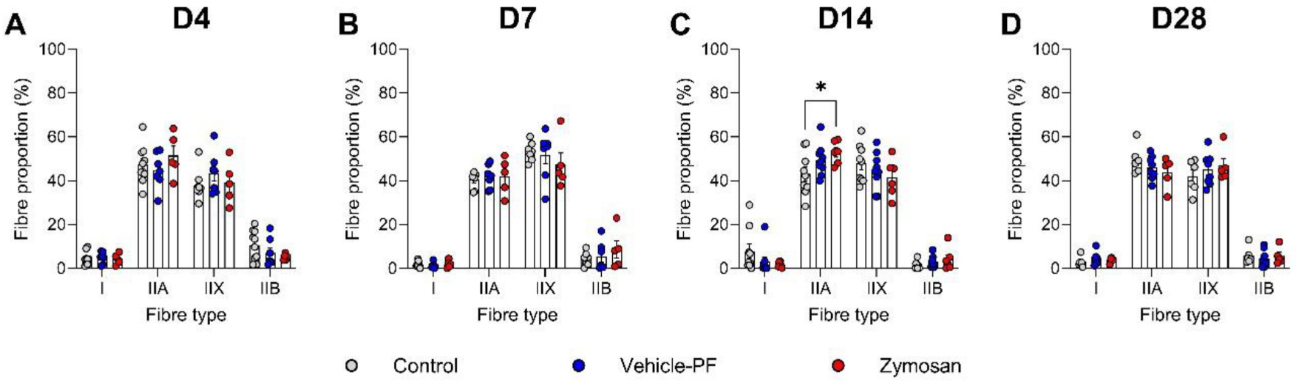

Fig. S15. Analysis of fibre proportion and fibre numbers in the diaphragm muscles of mice after zymosan administration. C57BL/6J male mice (15-16 wks) were allocated to control, vehicle or zymosan groups, with samples collected at 4, 7, 14 and 28 days after critical illness induction. Fibre proportions at D4 (A), D7 (B), D14 (C) and D28 (D) after critical illness. PF: pair-fed. Data are mean ± SEM, n=6-10/group. *P<0.05.

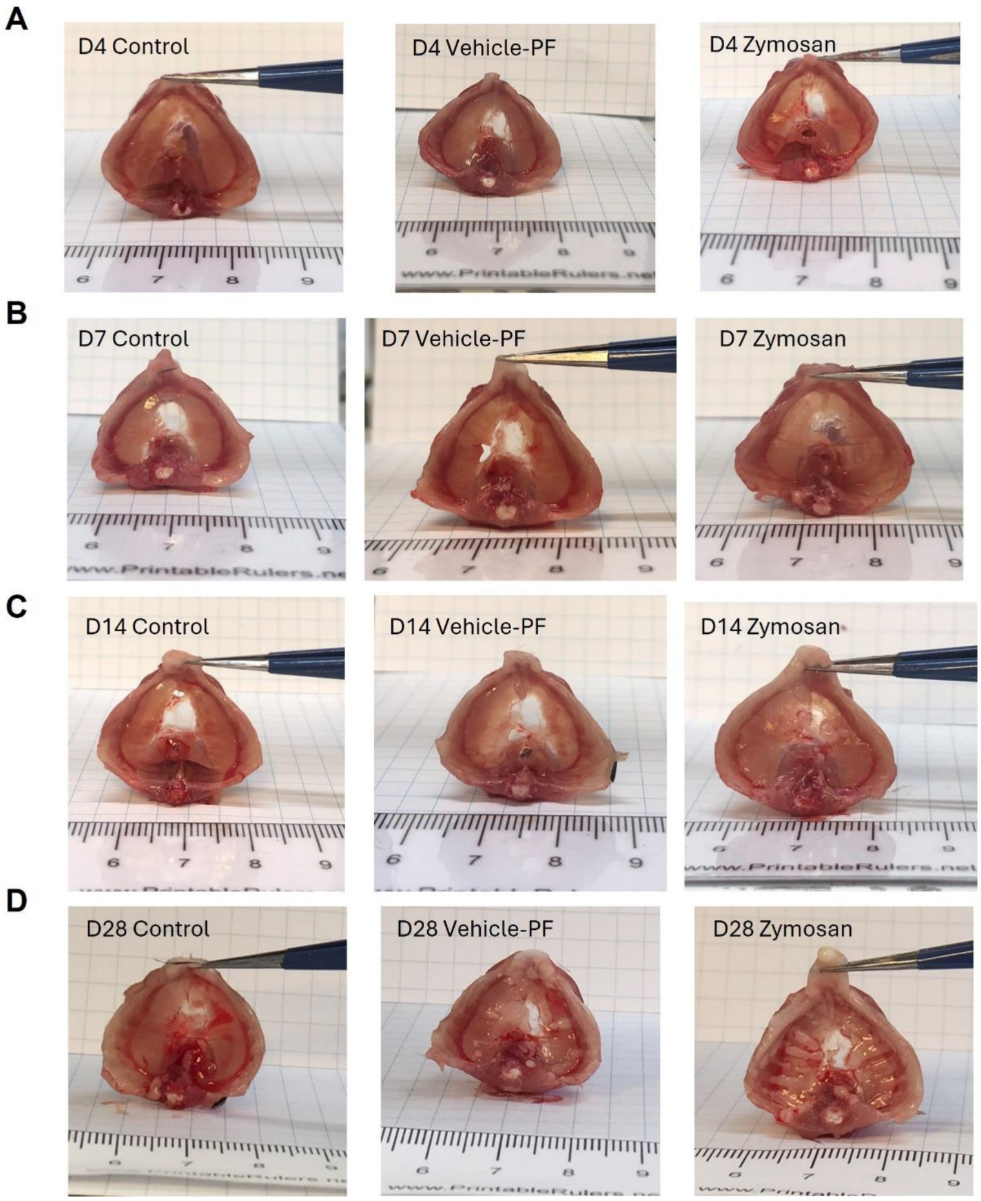

Fig. S16. Microgranulomas adhered to the abdominal side of the diaphragm in mice after zymosan administration. The appearance of microgranulomas (clusters of immune cells) worsened over the experimental period. Microgranulomas were not present at D4 (A) or at D7 (B) but began adhering to the diaphragm from D14 in zymosan treated mice (C). They were most prominent at D28 in zymosan treated mice and found (although less extensively) in vehicle treated mice at this timepoint (D). Representative images of the abdominal side of the diaphragm with rib cage intact. PF: pair- fed. Scale in in centimetres.

Disease Models & Mechanisms • Supplementary information

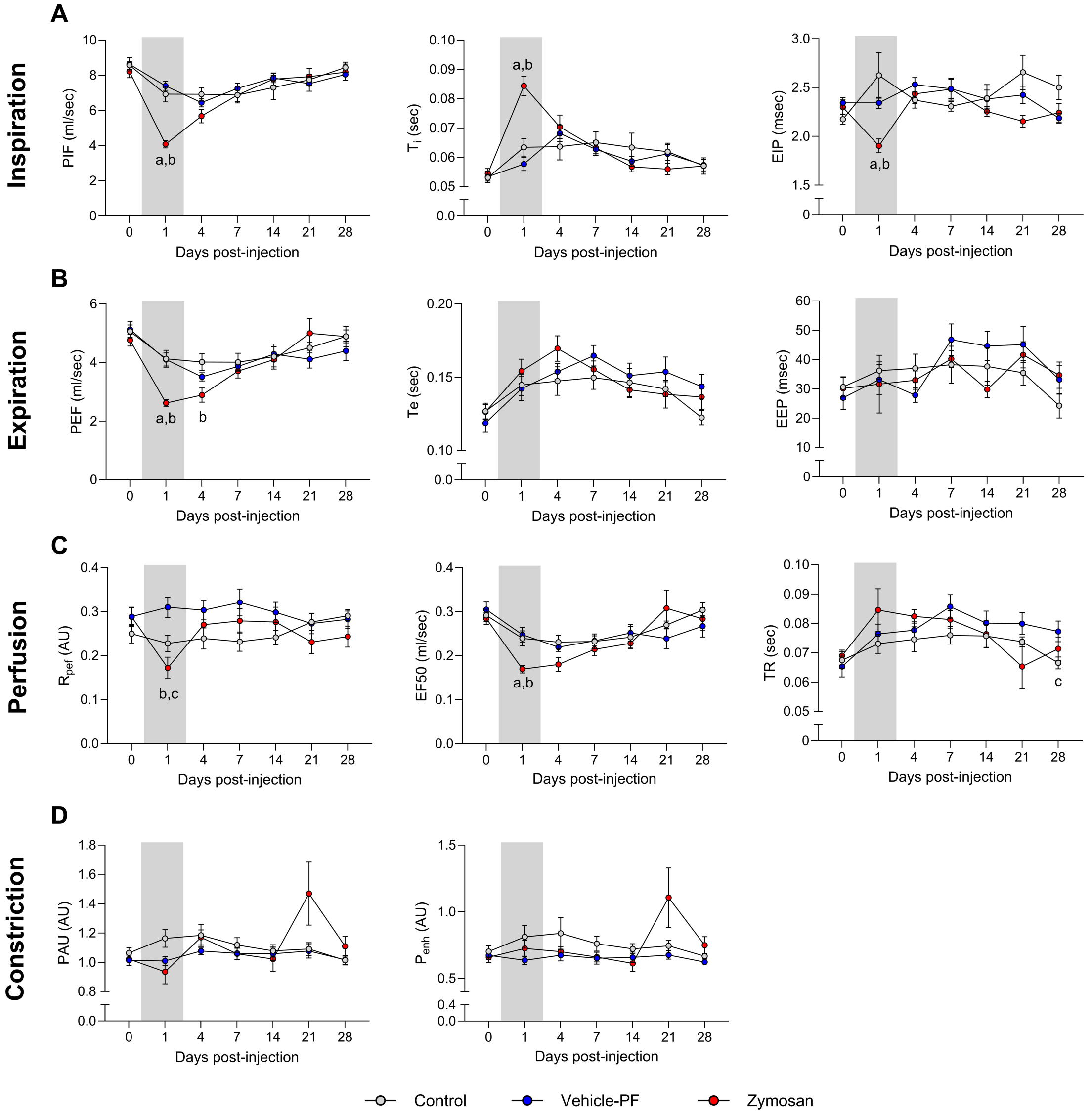

Fig. S17. No functional deficits in respiratory function in mice after zymosan administration based on whole body plethysmography. Respiratory dysfunction was examined at D-1 (baseline), D1, D4, D7, D14, D21, and at D28 after induction of critical illness. Inspiratory parameters (A), including Inspiratory flow (PIF; left panel), inspiratory time (Ti; middle panel) and end inspiratory pause (EIP; right panel). Expiratory parameters (B), including expiration (PEF; left panel), expiration time (Te; middle panel) and end expiratory pause (EEP; right panel). Perfusion parameters, including rate of perfusion (Rpef; left panel), uncompensated expiratory flow (EF50; 50% expired volume, right panel) and relaxation time

(TR; right panel). Lastly, constriction parameters (D), including pause (PAU; left panel) and enhanced pause, a marker of bronchoconstriction, were measured (Penh; right panel). AU: arbitrary units, PF: pair-fed. Data are mean ± SEM, n=8-9/group. [a]P<0.05 zymosan vs. vehicle, [b]P<0.05 zymosan vs. control, [c]P<0.05 vehicle vs. control.

Disease Models & Mechanisms • Supplementary information

Table S1. Muscle and organ masses in vehicle-treated mice fed ad libitum

	Control	Vehicle
**Tibialis anterior**	2.85±0.11	2.89±0.13
**Extensor digitorum longus**	0.65±0.02	0.66±0.01
**Rectus femoris**	5.65±0.08	5.39±0.13
**Soleus**	0.48±0.004	0.48±0.02
**Plantaris**	1.01±0.04	0.95±0.03
**Gastrocnemius**	7.67±0.13	7.34±0.18
**Heart**	7.01±0.23	6.79±0.12
**Spleen**	3.56±0.09	4.11±0.49
**Liver**	79.98±3.31	71.00±2.50
**Kidney**	9.22±0.46	7.62±0.77

Mice received an injection of liquid paraffin (vehicle) or were allocated to control (no treatment and fed *ad libitum* until experimental endpoint (day 4). Data are raw values (mg) normalised to tibia length (mm) with mean ± SEM (n=3/ group).

Table S2. Primary antibodies for assessment of inflammatory markers

Primary antibody	Dilution	Company	Catalogue no.	RRID
CD45	1:100	CST	55307	AB_3712912

CST: Cell Signalling Technologies (Danvers, MA, U.S.A).

Table S3. Secondary antibodies for assessment of inflammatory markers

Primary antibody	Secondary antibody				
	Antibody	Dilution	Company	Catalogue no.	RRID
CD45	AF647 GARat IgG	1:250	Invitrogen	A21247	AB_141778
	AF488 GAMouse IgG	1:250	Invitrogen	A11001	AB_2534069
	DAPI	1:1000	Invitrogen	D1306	-
N/A	AF647-Anti-CD68 conjugate	1:200	Abcam	ab201845	-
	DAPI	1:1000	Invitrogen	D1306	-

Invitrogen (Thermo-Fisher, Waltham, MA, U.S.A). DAPI: 4′,6-diamidino-2-phenylindole, GA: Goat-anti.

Table S4. Primary antibodies for fibre type analyses

Primary antibody	Dilution	Company	Catalogue no.	RRID
Anti-Laminin	1:200	Sigma-Aldrich	L9393	AB_477163
MyHC I (BA-D5*)	1:25	DSHB	-	AB_2235587
MyHC IIA (SC-71*)	1:25	DSHB	-	AB_2147165
MyHC IIB (BF-F3*)	1:10	DSHB	-	AB_2266724

MyHC: Myosin Heavy Chain. DSHB: Developmental Studies Hybridoma Bank. *Antibodies developed by S. Schiaffino, University of Padova (Padua, Italy).

Table S5. Secondary antibodies for fibre type analyses

Primary antibody	Secondary antibody				
	Antibody	Dilution	Company	Catalogue no.	RRID
**Tibialis anterior and rectus femoris sections**					
Anti-Laminin	AF555 GAR IgG	1:250	Invitrogen	A21428	AB_2535849
MyHC I (BA-D5)	AF350 GAM IgG2b	1:250	Invitrogen	A21140	AB_2535777
MyHC IIA (SC-71)	AF647 GAM IgG1	1:250	Invitrogen	A21240	AB_2535809
MyHC IIB (BF-F3)	AF488 GAM IgM	1:250	Invitrogen	A21042	AB_2535711
**Soleus and diaphragm sections**					
Anti-Laminin	AF555 GAR IgG	1:250	Invitrogen	A21428	AB_2535849
MyHC I (BA-D5)	AF488 GAM IgG2b	1:250	Invitrogen	A21141	AB_2535778
MyHC IIA (SC-71)	AF647 GAM IgG1	1:250	Invitrogen	A21240	AB_2535809
MyHC IIB (BF-F3)	AF350 GAM IgM	1:250	Invitrogen	A31552	AB_2536169

AF: Alexa Fluor, MyHC: Myosin Heavy Chain, GAR: Goat-anti rabbit, GAM: Goat-anti mouse.

Disease Models & Mechanisms • Supplementary information
